# *Pseudomonas aeruginosa* Genome Evolution in Patients and under the Hospital Environment

**DOI:** 10.3390/pathogens3020309

**Published:** 2014-04-10

**Authors:** Céline Lucchetti-Miganeh, David Redelberger, Gaël Chambonnier, François Rechenmann, Sylvie Elsen, Christophe Bordi, Katy Jeannot, Ina Attrée, Patrick Plésiat, Sophie de Bentzmann

**Affiliations:** 1 Genostar, 60 rue Lavoisier, Montbonnot 38330, France; E-Mails: miganeh@genostar.com (C.L.-M.); rechenmann@genostar.com (F.R.); 2 UMR7255-Laboratoire d’Ingénierie des Systèmes Macromoléculaires, CNRS—Aix Marseille University, Marseille 13402, France; E-Mails: redel@imm.cnrs.fr (D.R.); gchambonnier@imm.cnrs.fr (G.C.); bordi@imm.cnrs.fr (C.B.); 3 INSERM, UMR-S 1036, Biology of Cancer and Infection, Grenoble 38054, France; E-Mails: sylvie.elsen@cea.fr (S.E.); ina.attree-delic@cea.fr (I.A.); 4 Laboratoire de Bactériologie, Faculté de Médecine-Pharmacie, Université de Franche-Comté, Besançon 25030, France; E-Mails: katy.jeannot@univ-fcomte.fr (K.J.); patrick.plesiat@univ-fcomte.fr (P.P.)

**Keywords:** *Pseudomonas aeruginosa*, microevolution, genome, virulence, resistance, cystic fibrosis, ventilator-associated pneumonia, ICU

## Abstract

*Pseudomonas aeruginosa* is a Gram-negative environmental species and an opportunistic microorganism, establishing itself in vulnerable patients, such as those with cystic fibrosis (CF) or those hospitalized in intensive care units (ICU). It has become a major cause of nosocomial infections worldwide and a serious threat to Public Health because of overuse and misuse of antibiotics that have selected highly resistant strains against which very few therapeutic options exist. Herein is illustrated the intraclonal evolution of the genome of sequential isolates collected in a single CF patient from the early phase of pulmonary colonization to the fatal outcome. We also examined at the whole genome scale a pair of genotypically-related strains made of a drug susceptible, environmental isolate recovered from an ICU sink and of its multidrug resistant counterpart found to infect an ICU patient. Multiple genetic changes accumulated in the CF isolates over the disease time course including SNPs, deletion events and reduction of whole genome size. The strain isolated from the ICU patient displayed an increase in the genome size of 4.8% with major genetic rearrangements as compared to the initial environmental strain. The annotated genomes are given in free access in an interactive web application WallGene designed to facilitate large-scale comparative analysis and thus allowing investigators to explore homologies and syntenies between *P. aeruginosa* strains, here PAO1 and the five clinical strains described.

## 1. Introduction

Genome sequencing capabilities have expanded exponentially in the last 10 years and the number of finished or draft genomes has considerably enriched databases [1]. This growing bulk of data has already proven to be useful for strain-to-strain comparisons, to obtain novel information on pathogen diversity, as well as to rapidly identify bacterial genes that play a role in infection and SNPs or other mutational events that shape the virulence in clonal expansion of strains. Several sequencing techniques are now available at low cost that can address all these issues. However, access to bioinformatic tools that rapidly provide a user-friendly way to compare neo-sequenced genomes with references available in databases is required for microbiologists.

*Pseudomonas aeruginosa*, a Gram-negative environmental species and an opportunistic pathogen, is a major cause of infections in vulnerable patients with cystic fibrosis (CF) or admitted to intensive care units (ICUs). With ca. 10% nosocomial infections incurred, this pathogen is now considered as serious problem to Public Health worldwide. In September 2013, the US Centers for Disease Control and Prevention (CDC) announced that the threat level associated with *P. aeruginosa* was “serious” because of high number of untreatable infections. Thus, an expanding number of genome sequencing projects have been launched to unravel the complex genetic changes occurring in *P. aeruginosa* during long-term chronic infections such as those encountered in CF [2,3,4,5,6,7,8,9] or to investigate the multiple resistance determinants of ICU strains [10]. In the present work, we sequenced and annotated genomes of *P. aeruginosa* isolates sequentially collected from a single CF patient as well as of two clonally-related isolates recovered in a same ICU (sink and patient) but having several different phenotypic traits. Draft genome versions were obtained by using Illumina Hiseq 2000 system, Genostar suite software and WallGene facilities allowing to track possible genetic events that could sustain their phenotypes. The clinical strains were compared to reference strain PAO1. Applied to CF (chronic evolution) and ICU (fast evolution) *P. aeruginosa* strains, our data provide new insights into the genomic diversification of clones, the potential role of prophages in shaping bacterial genomes in specific environments and key interesting genetic or genomic events that point out mechanisms that need further investigation. 

## 2. Results and Discussion

### 2.1. Strains

#### 2.1.1. Description of KK Related-Strains

Three related isolates, named KK, belonging to a same lineage A were sequentially collected from the sputum samples of a CF patient followed at the CF clinic of Hannover (Germany) who exhibited a *cftr* ΔF508/ΔF508 genotype associated with an exocrine pancreatic insufficiency.

The KK1 strain corresponds to the primo-colonization strain isolated when the patient was 16.2 year-old, the second KK14 strain was isolated 18 months later, and the third strain KK72 was recovered 156 months after the KK1 strain. The patient died shortly after this latest sampling.

##### 2.1.1.1. Growth Characteristics

**Figure 1:**
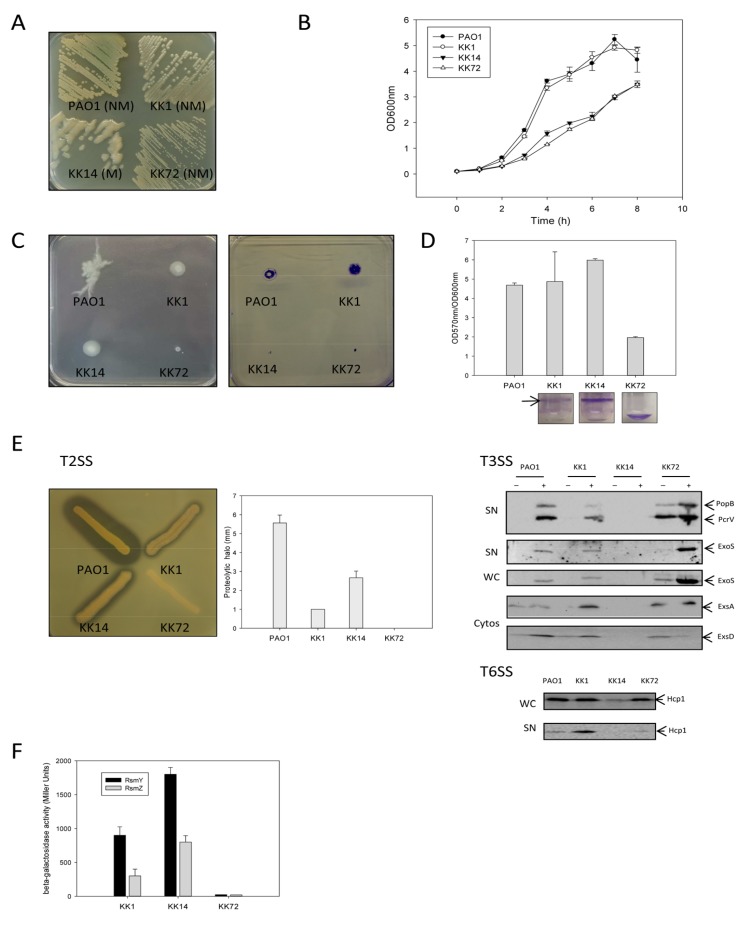
Phenotypic characterization of KK cystic fibrosis (CF) strains. From the top left to the bottom right panels: (**A**) Aspect of KK strains plated on PIA agar plates (M for mucoid aspect and NM for non mucoid aspect); (**B**) Growth curves of KK strains in LB medium at 37 °C (n = 3); (**C**) Swarming and twitching behaviors of KK strains; (**D**) Biofilm biomass formed by KK strains evaluated by OD_570 nm_ measurement normalized by the OD_600 nm_ (growth) after crystal violet staining; (**E**) Proteolytic activity (highly dependent on T2SS) of extracellular products produced by KK strains. The mean and standard deviation of proteolytic halo measurements were presented (n = 9); T3SS production of KK strains evaluated in non induced or induced conditions (Ca^2+^ chelation) by the synthesis and secretion of the translocon proteins PopB and PcrV of the needle apparatus and the exotoxin ExoS, the cytosolic (Cytos) production of the positive regulator protein ExsA of the *P. aeruginosa* T3SS regulon [14] and of the anti-ExsA protein ExsD [15]; T6SS (HSI-1) production of KK strains evaluated by the synthesis (WC) and secretion (SN) of the Hcp1 protein; (F) Activity of *rsmY* and *rsmZ* promoters in KK strains.

Under laboratory conditions, strains KK1 and PAO1 grew better than KK14 and KK72 (Figure 1B). Only KK14 produced mucoid colonies, a phenotype associated with alginate production (Figure 1A). 

##### 2.1.1.2. Motility and Biofilm Capacities

In addition to being mucoid, KK14 is non-flagellated and known to promote a higher IL6 and TNFα release from monocytes as compared to PAO1 reference strain [11]. None of the KK strains displayed a swarming phenotype, and the twitching motility of KK1 strain was identical to that of PAO1, whereas KK14 and KK72 did not twitch at all (Figure 1C). Under static growth conditions *in vitro*, KK1 and KK14 formed biofilm while KK72 did not (Figure 1D).

##### 2.1.1.3. Proteolytic Activity, T3SS and T6SS

We previously showed that KK1 strain produced a phosphate limitation-like secretome [12] very different from the PAO1 one, including production of the Type 6 secretion system (T6SS HSI-I) marker protein Hcp1, that is part of the cell-puncturing device allowing the translocation of effectors into other bacterial species [13], but lacking T2SS-dependent substrates such as elastase. Proteolysis halo observed on TSA milk plates mainly indicating recovery of LasB in the extracellular medium, was strongly and statistically attenuated in KK strains as compared to PAO1, although KK14 displayed the highest activity among the KK strains (Figure 1E). Proteolytic halos were significantly different in size between KK strains.

Type 3 secretion system (T3SS) is a secretion machinery looking like a molecular needle able to deliver cytotoxic effectors into host cells and thus which initiates and maintains infection by manipulating host cell biology such as cell signaling, secretory trafficking, cytoskeletal dynamics, and the inflammatory response (for review, see [16,17,18]). Clearly the KK strains were not equivalent T3SS producers. While KK1 produced and secreted T3SS proteins (here, the translocator proteins PopB and PcrV, and the effector protein ExoS) under calcium-depleted conditions similarly to PAO1, KK14 did not produce any of the T3SS proteins under both T3SS-induced and non-induced conditions. The transcriptional activator ExsA and its anti-activator, the ExsD protein were not detected in the KK14 strain, reflecting a strong defect or lack of T3SS gene expression. On the contrary, increased synthesis of ExoS and PopB/PcrV was observed in KK72 whatever the growth conditions used compared to KK1, in accordance with upregulated synthesis of ExsA even under high calcium concentrations. High amounts of ExoS were present in the supernatant of calcium-depleted cells, while PopB and PcrV appeared to be secreted under both conditions, in agreement with the absence of calcium control on translocator secretion [19] (Figure 1E). Transcription of genes (data obtained from transcriptomes of KK strains) encoding T3SS machinery and regulators as well as T3SS effectors was in complete agreement with our translation data (not shown).

T6SS production was effective in KK1 [12], strongly reduced in KK14, and similar to KK1 and PAO1 in KK72. Recovery of Hcp1 in supernatants was maximal in KK1, attenuated in KK72 and PAO1, and undetectable in KK14 (Figure 1E).

##### 2.1.1.4. sRNA Rsm

The mutually exclusive biofilm-T3SS phenotypes, hallmarks of small non-coding RNA (sRNA) RsmY and RsmZ-dependent regulation [20,21] were investigated by monitoring the levels of *rsmY* and *rsmZ* transcriptional fusions. The *rsmY* and *rsmZ* promoter activities were found to be the highest in KK14, at intermediate level in KK1, and under the detection limit in KK72 (Figure 1E). Unexpectedly, T6SS production in KK strains (and transcript levels as well, data not shown) were not correlated with Rsm sRNA levels as already reported in other studies, suggesting that in KK strains, another level of T6SS regulation is operating that should be examined in the future.

#### 2.1.2. Description of ST395 Related-Strains

Two genotypically identical isolates belonging to an epidemic clone, ST395, previously implicated in hospital outbreaks [22] were isolated from a sink (ST395E) and from a mechanically ventilated patient (ST395P) in a same intensive care unit (ICU) at the teaching Hospital of Besançon (France).

##### 2.1.2.1. Growth Characteristics

**Figure 2:**
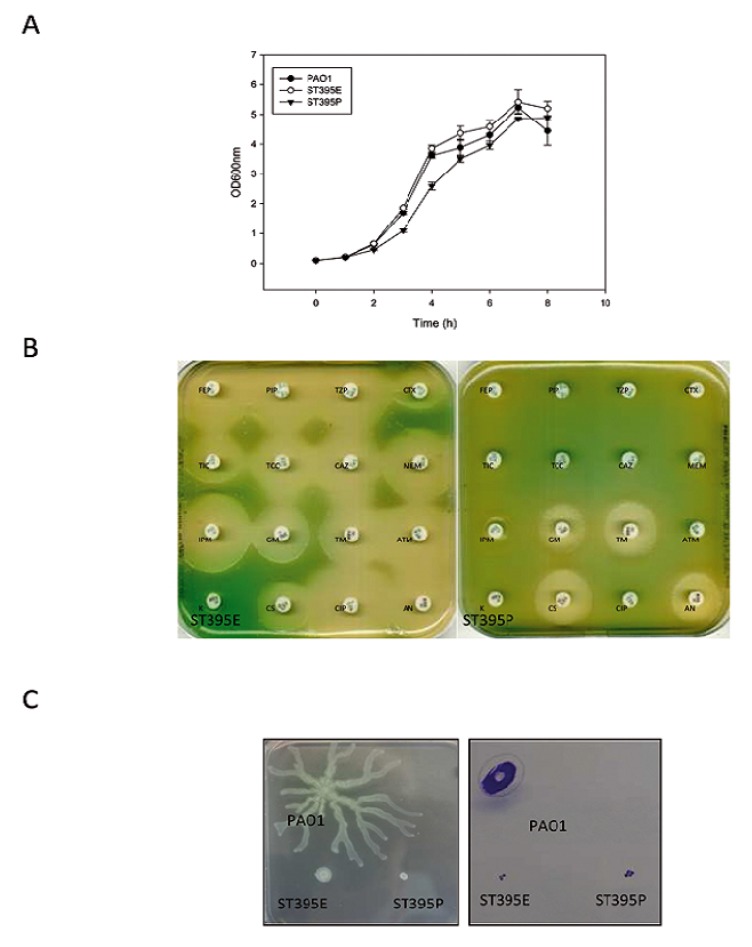
Phenotypic characterization of ST395 ICU-related strains. (**A**) Growth curves of the ST395 strains in LB medium at 37 °C (n = 3); (**B**) Antibiograms of ST395 strains (FEP: cefepim, PIP: piperacillin, TZP: piperacillin+tazobactam, CTX: cefotaxime, TIC: ticarcillin, TCC: ticarcillin+clavulanic acid, CAZ: ceftazidime, MEM: meropenem, IPM: imipenem, GM: gentamicin, TM: tobramycin, ATM: aztreonam, K: kanamycin, CS: colistin, CIP: ciprofloxacin, AN: amikacin); (**C**) Swarming behaviour of ST395 strains grown on M8 medium supplemented with 0.5% agar for 48 h at 37 °C; Twitching behavior of ST395 strains grown on LB medium supplemented with 1.5% agar for 48 h at 37 °C.

The environmental isolate ST395E grew slightly faster than the clinical one, ST395P, (Figure 2A) though the difference was minimal after 8 h incubation. 

##### 2.1.2.2. Antibiotic Resistance

While ST395E was susceptible to antipseudomonal antibiotics, ST395P exhibited panresistance to ß-lactams (including carbapenems imipenem and meropenem, penicillins ticarcillin ± clavulanic acid and piperacillin ± tazobactam, cephalosporins ceftazidime, cefepime, and cefotaxime, monobactam aztreonam) and fluoroquinolones (ciprofloxacin) (Figure 2B). A low level of resistance to aminoglycosides (amikacin, tobramycin and gentamicin) was also noted while ST395P remains susceptible to colistin thus fitting the definition of extremely drug resistance (XDR) [23]. Both ST395 strains turned out to be defective in swarming and twitching motility (Figure 2C).

### 2.2. Genome Examination

We obtained the assembled draft genomes of these five strains and compared them with reference strain PAO1, a primary analysis that will be completed by using other published annotated genomes as comparators [24].

**Figure 3:**
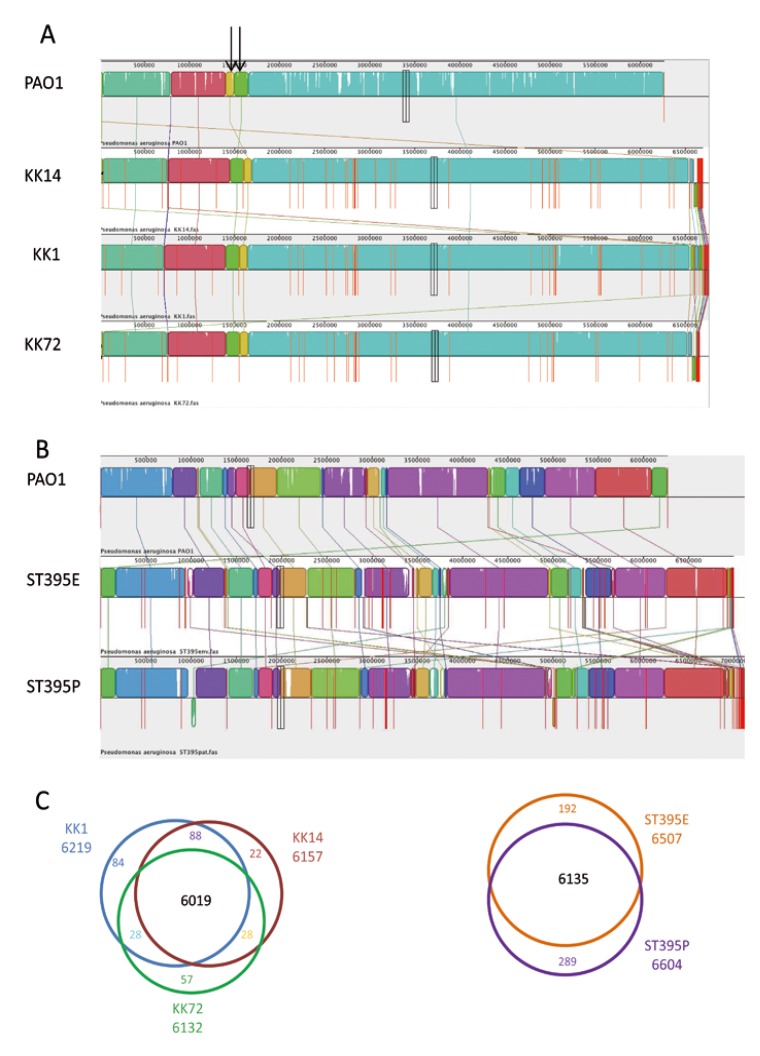
Alignment of genomes using MAUVE. Pairwise alignment between the three KK strains (**A**) and the ST395 strains (**B**) and the complete genome of *P. aeruginosa* PAO1 using the MAUVE software. Colored blocks outline genome sequences that align to part of another genome, and is presumably homologous and internally free of genomic rearrangement (Locally Colinear Blocks or LCBs). White regions correspond to sequences that are not aligned and probably contain sequence elements specific to a particular genome. Blocks below the center line indicate regions that aligned in the reverse complement (inverse) orientation. The height of the profile within each LCB demonstrates the average degree of sequence conservation within an aligned region; (**C**) Venn diagrams of KK and ST395 strains.

We investigated the overall genomic differences between PAO1 and KK strains by aligning the genomes using MAUVE 2.3.1 software (Figure 3A). Alignment of the assembled contig sequences of draft genomes of KK strains with PAO1 suggests a high level of conservation along the chromosome. The KK strains have their chromosomes organized in a very similar way than in PAO1 except one inversion of two physically close syntenic blocks colored in green and yellow in Figure 3A (highlighted by arrows). Based on the total length of KK assembled contig sequences, as well as on the size of the PAO1 genome (6,264,404 bp), we could estimate the percentages of genome covered to be 97% for KK1 and KK14, and 96.6% for KK72. KK1 has an estimated genome size of 6,759,575 bp (6219 ORF with a GC% of 63.7), KK14 of 6,690,898 bp (6,157 ORF with a GC% of 64.1) and KK72 of 6,657,327 bp (6132 ORF with a GC% of 63.9) (Table 1), suggesting a slight reduction in the genome size during the pulmonary colonization timescale.

The draft genomes of the three KK strains were compared with that of PAO1 using the WallGene software. KK1, KK14 and KK72 were thus found to share 5308 genes with PAO1 and to contain 911,849,824 specific genes in their accessory genome, respectively. The KK strains shared a core of 6019 genes and had 84, 22 and 57 genes of difference between them, respectively (Figure 3C), while 222, 225 and 251 genes of PAO1 were absent in KK1, KK14 and KK72, respectively.

We investigated the overall genomic differences between PAO1 and ST395 strains by aligning the three genomes using the MAUVE 2.3.1 software (Figure 3B). Based on the total length of ST395 assembled contigs, as well as on the size of the PAO1 genome, we could estimate the percentages of genome covered to be 97.5% and 97.7% for ST395E and ST395P genomes, respectively. ST395E has a genome size of 6,993,173 bp (6,507 ORF with a GC% of 65.9) while ST395P has a genome of 7,133,660 bp (6,604 ORF with a GC% of 63.1) (Table 2).

The draft genomes of these two ICU strains were compared with that of PAO1 using WallGene. From these results, it appeared that ST395E and ST395P shared 5322 genes with PAO1 but contained 1185 and 1282 additional accessory genes, respectively. ST395 strains had 6135 genes in common but differed by 192 and 289 specific genes, respectively (Figure 3C). On the other hand, 189 and 181 genes present in PAO1 were lacking in ST395E and ST395P genomes, respectively. We next focused our analysis on regions of genome plasticity (RGP) in KK and ST395 strains.

#### 2.2.1. KK Strain Microevolution and Phenotypic Consequences

The regions differing in KK strains as compared to PAO1 strain but also, changes that have occurred in the different KK genomes (summarized in Table 3 and Figure S1) were further examined.

**Table 1:** Statistics and features of the sequenced *P. aeruginosa* KK strains.

Strains	PAO1	KK1	KK14	KK72
Numbers of reads	NA	10,357,778	10,357,778	10,357,778
Average read length (bp)	NA	90	90	90
Sequence coverage	NA	138	140	140
DNA scaffolds	NA	95	78	125
DNA total number bases	6,264,404	6,759,575	6,690,898	6,657,327
ORF	5571	6219	6157	6132
Genes with EC number (enzymes)	NA	978	978	974

NA: Non available.

**Table 2:** Statistics and features of the sequenced *P. aeruginosa* ST395 strains.

Strains	PAO1	ST395E	ST395P
Numbers of reads	NA	9,422,224	9,880,000
Average read length	NA	90	90
Sequence coverage	NA	121	125
DNA scaffolds	NA	55	86
DNA total number bases	6,264,404	6,993,173	7,133,660
ORF	5571	6507	6604
Genes with EC number (enzymes)	NA	982	982

NA: Non available.

**Table 3:** Regions of genome plasticity of the sequenced *P. aeruginosa* KK strains.

	Location in PAO1 genome	Phagic origin	KK1	KK14	KK72
Region KK_1	PA3357-PA3391 (24 kb)	no	+ (33.119 kb)	+ (33.119 kb)	-
Region KK_2	PA0632-PA0649	yes	-	-	-
Region KK_3 Prophage KK_1	PA0729.1	yes ΦCTX	+ (38.859 kb)	+ (38.859 kb)	-
Region KK_4 Prophage KK_2	PA0820-PA0826.1	yes F10	+ (>54.093 kb)	+ (>54.074 kb)	+ (>54.093 kb)
Region KK_5	PA1087-PA1094	no	+ (12.703 kb) Novel locus	+ (12.703 kb) Novel locus	+ (12.703 kb) Novel locus
Region KK_6 Prophage KK_3	PA1796.1-PA1796.4	yes Φ297	+ (51.617 kb)	-	+ (51.625 kb)
Region KK_7 Prophage-like_KK_4	PA4673.1	??	+ (>24.424 kb)	+ (22.616 kb)	+ (24.664 kb)
Region KK_8	PA2593-PA2594	no	+ (31.824 kb)	+ (34.971 kb)	+ (31.814 kb)
Region KK_9	PA2819.1-PA2819.3	no	+ (123.358 kb)	+ (122.554 kb)	+ (122.566 kb)
Region KK_10	PA2583.1	no	+ (102.482 kb)	+ (>83.975 kb)	+ (102.482 kb)
Region KK_11	PA3768-3769	no	+ (6.955 kb)	+ (6.955 kb)	+ (6.955 kb)
Region KK_12	Upstream PA2077	no	+ (57.2 kb)	+ (57.2 kb)	+ (57.2 kb)

##### 2.2.1.1. Major Genomic Changes between KK Strains

A region consisting of 33 genes, named KK_1, was present in the KK1, KK14 and PAO1 genomes but absent in KK72. In KK1 and KK14, this locus contains additional ORF genes covering approximately 8 kb, compared with PAO1.

The region named KK_2 of PAO1 genome which overlaps the RGP4 previously identified (PA0641-PA0648) [25] was absent in all KK strains, at least at that location and in the covered sequences.

**Figure 4:**
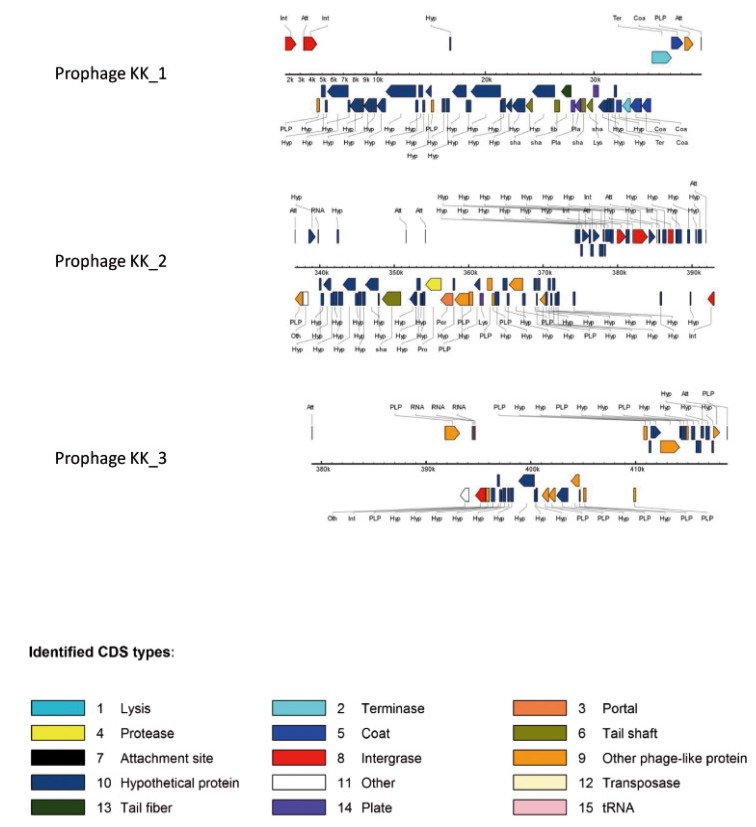
Prophages identified in KK strains. The identification has been performed using PHAST software; each arrow represents a gene predicted by PHAST and color codes indicate the corresponding functions of the genes.

The region KK_3 of 38,859 kb (prophage KK_1 corresponding to 50 ORF with a GC% of 61.4, Figure 4) was identified in KK1 and KK14 strains but not in KK72 strain. This region displays high homology with bacteriophage ΦCTX.

The region KK_4 of 56.3 kb (prophage KK_2 corresponding to 81 ORF with a GC% of 60.6, Figure 4) replaced the locus PA0820-PA0826.1 of PAO1 in all the KK strains. This region displays a high homology with bacteriophage F10 although the full region was unavailable in all the KK strains due to overlapping of this region with ends of contigs.

The 12.3 kb-long region KK_5 located between PA1087 (*flgL*) and PA1094 (*fliD*), that in the PAO1 genome contains the *fgtA* and the *fliC* genes encoding the flagellar glycosyltransferase FgtA and the type B flagellin, respectively, was found to be replaced in all the KK strains by a locus of 9 genes identical to RGP9 of strain PAC2, encoding among others, a type A flagellin [25].

The region KK_6 (prophage KK_3 corresponding to 38 ORF with a GC% of 61.1, Figure 4) was identified in KK1 and KK72. This region displays high homology with bacteriophage Φ297. In KK14, the prophage sequence was missing except 2 genes indicating a partial excision.

The region KK_7 (prophage-like KK_4 corresponding to 38 ORF with a GC% of 61.1) occurring in all the KK strains appeared to be incomplete since present at the ends of contigs in all these bacteria. PHAST software was unable to ascertain the phage origin of this region, probably because of incomplete sequences of the draft genomes.

Region KK_8 (31.5 kb, 20 ORF) was present in all the KK strains while absent from PAO1. This region carries the *pltLABCDEFGMR* locus involved in biosynthesis of antifungal product pyoluteorin, a hybrid polyketide-nonribosomal peptide molecule of *Pseudomonas fluorescens* Pf-5 [26] also produced by LESB58 and M18 strains of *P. aeruginosa.* Whether it contributes to antifungal defence in the CF lungs has to be elucidated.

Region KK_9 (125 kb) was found to be inserted in all the KK strains between gene PA2818 encoding the aminoglycoside response regulator Arr [27] and the PA2820 gene in place of the tRNA-Gly, Gly and Glu PA2819.1 to PA2819.3 of the PAO1 strain. KK_9 is part of the RGP29 identified in PA2192 strain [25] (from PA2G_02184 to PA2G_02073) with particularities of this region in KK strains since it possesses a gene encoding a supplementary heavy metal RND efflux pump of the CzcABC type, several genes encoding putative copper resistance proteins and putative regulatory proteins.

The region KK_10 of 106 kb in length detected in all the KK strains between PA2583 and PA2584 in place of the tRNA-Gly PA2583.1 of PAO1 genome exhibited high homologies with the PA_01003088 to PA_01003140 genes of RGP27 of from *P. aeruginosa* strain PAC2 strain [25]. This region was incomplete in KK14 since ending a contig (Figure S1).

A region of 6 genes, named KK_11, was present in all the KK strains in between PA3768 and PA3769 (*guaA*), of which 3 genes had homologs (PA14_15620 to PA14_15650) in RGP36 of PACS2 or PA14 strains [25].

Finally, the KK strains all displayed a 38 gene locus (region KK_12, 57.2 kb) located between the PA2077 and PA2078 genes of PAO1, encoding proteins involved in mercuric resistance (with *merE*, *merD*, *merA*, *merP*, *merR* and *merT* homologs [28]), corresponding to an RND multi-drug efflux pump and a regulator of EAL family.

All phage regions were confirmed by mapping reads and therefore we could confirm that when stated these regions are missing.

##### 2.2.1.2. Minor Genomic Changes between KK Strains

Regarding additional genomic DNA differences between KK strains and PAO1, more discrete remodeling events were noticed. For instance, in KK strains a gene encoding a putative acetyltransferase was found inserted downstream the *pdxY* (PA5516) gene compared to PAO1. Similarly, an additional gene encoding an asparagine synthetase was identified downstream the *rfaE* (PA4996) gene of PAO1, in all the KK strains just as in *P. aeruginosa* M18. The gene encoding AlgP, the prokaryotic transcriptional positive regulator required for transcription of key alginate biosynthetic gene *algD*, contained in all the KK strains an extra 12-bp repeated sequence corresponding to a KPAA additional module as compared to PAO1. However, although the repetitive structure of the *algP* gene appears to participate in the processes underlying the metastable character of mucoidy in *P. aeruginosa*, variations in the number of the 12-bp repeats found did not appear to influence the mucoid status of the examined strains [29].

On the other side, several specific regions of PAO1 were not identified in the KK strains. As mentioned previously, this was the case of region KK_2 and of three ORF (PA3486-PA3488) including *pldA* and *vgrA1* genes which encodes a phospholipase D and an effector of the HSI-I T6SS, respectively. These later genes are present on a 7 kb mobile genetic element acquired horizontally, perhaps from an eukaryotic organism, of which *pldA* has been demonstrated to contribute to the ability of *P. aeruginosa* PAO1 to persist in a chronic pulmonary infection model in rats [30]. The region PA3497-PA3514 of PAO1 corresponding to RGP34 was absent from the KK strains as it is from strains PA14, PACS2, PA2192 and C3719 [25].

##### 2.2.1.3. Genetic Changes Related to Evolution of Phenotypic Traits of KK Strains over Time in CF Patient

**Figure 5:**
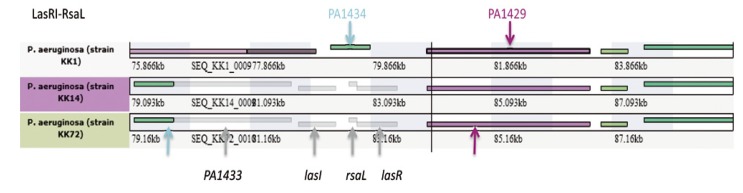
KK strain regions of genome plasticity (RGPs) varying between KK strains using WallGene facility. KK1 genome was chosen as the reference genome. The following color codes are used for genes in WallGene: (1) genes of the reference organism with a homologous gene in at least one of the non-reference genomes have an attributed color (except black, grey, or white) at random; (2) genes of the reference genome with no homology at all are black; (3) genes of non-reference genomes with exactly one homology in the reference genome are the same color as the homologous gene; (4) genes of non-reference genome with more than one homology in the reference genome are white; (5) genes of non-reference genomes with no homology in the reference genome are dark grey. If homology links are displayed, those genes are hidden.

*Quorum Sensing and Alginate*. We previously demonstrated that KK1 strain produces a phosphate limitation-like secretome very different from the PAO1 one, in particular lacking the elastase LasB [12]. We thus examined what could explain this particular secretory phenotype. Interestingly, this strain lacks the PA1430-1433 genes encoding the LasR-LasI quorum sensing cell-cell communication system that controls expression of many exoproducts in *P. aeruginosa* [31], the RsaL negative regulator of LasR-LasI system [32] and a protein of unknown function (Figure 5). Interestingly, KK14 and KK72 strains have this locus. Moreover, LasR of KK72 strain displays a K218R substitution (Figure 8A) in the C-terminal part of this LuxR type regulator forming an HTH domain contacting DNA [33]. The impact of this amino-acid substitution in the α9 helix critical for DNA recognition [34] and for binding of the LasR transcriptional activator in KK72 strain requires further examination. These genetic changes seem to correlate well with the global analysis of proteolytic activity of these strains (Figure 1E), although in depth analysis has to be performed. This suggests that the absence of this particular region could represent a fitness advantage for *P. aeruginosa* onset of infection in the KK environment.

KK1 and KK72 strains display a non mucoid phenotype, while KK14 strain exhibits a mucoid phenotype (Figure 1A). Examination of sequence of the *mucA* gene in KK strains identified a deletion of one G base in a stretch of 5G specific to KK14 strain already identified as a hot spot of mutation in *mucA* gene and leading to a frameshift [35]. The non mucoid phenotype of KK72 is associated with a wild type *mucA* gene. Transcriptomic data obtained from these three strains further confirmed the derepression of the *alg* biosynthetic genes in KK14 as compared to KK1 and KK72 strains (data not shown). Additionally, examination of variants showed that *algD* gene encoding a periplasmic epimerase which converts ß-D-mannuronic acid into α-L-guluronic acids at the polymer level of the alginate biosynthetis machinery [36] exhibits a SNP R401H in KK14 as compared to KK1 and KK72 strains. Whether this SNP contributes to KK14 mucoidy conversion remains to be elucidated.

*GacS/GacA regulatory pathway and related phenotypes*. We further examined KK genomes for the central GacS/GacA regulatory pathway controlling sRNA Rsm levels, since it has been demonstrated to be impaired in several clinical strains [5,37,38]. High expression of *rsmY* and *rsmZ* leads to massive biofilm formation and T6SS production and to repression of T3SS expression, while an impaired biofilm formation and no T6SS production and an induction of T3SS expression is associated with low or null levels of RsmY and RsmZ [39]. In *P. aeruginosa*, transcription of these two sRNAs is under a complex and sophisticated regulatory network involving the GacS/GacA two-component system (TCS) but also other TCS pathways including (i) the two histidine kinases (HK) LadS and RetS which triggers and represses expression of both *rsm* genes by interfering with the GacS/GacA TCS activity, respectively [40,41,42]; (ii) the GacS-structurally related PA1611 hybrid HK interacting with RetS in *P. aeruginosa* in a very similar manner than GacS and RetS do [43]; (iii) the HptB regulatory pathway which also intersects with the GacS/GacA TCS and induces only the expression of *rsmY* gene [39] and their associated HK PA2824 (SagS) [44] and PA1975 and iv/ the TCS BfiS/BfiR [45]. We thus checked if partners of the complex and sophisticated regulatory networking controlling Rsm production display or not mutations in KK strains. No mutation in LadS, GadS, GacA, RetS, PA1611 or PA1975, the regulator of PA1611, or in HptB was identified although these proteins are sharing polymorphism in all KK strains as compared to PAO1. No SNP was identified in promoter sequences of these genes or in *rsm* promoters between the three KK strains. Thus, this regulatory pathway remains functional during the period of isolation of KK strains, a feature interesting since LadS or GacS have been found to be susceptible to mutations in PA14 [38] or in the CF CHA [5,37] strains. TCS are not the only players involved in controlling *rsm* expression. Intracellular level of cyclic di-GMP has been described to control *rsm* expression [46] and an elevated intracellular concentration of cyclic di-GMP leads to an increased production of RsmY and RsmZ. This intracellular level of cyclic di-GMP is oppositely controlled by phosphodiesterases (PDE) and diguanylate cyclases (DGC) and any modification of the expression level of these proteins could thus lead to modification of *rsm* expression. Interestingly, KK14 strain does not produce and secrete T6SS components while forming biofilm and KK72 produces and secretes T6SS components while not forming biofilm, thus in these two strains, T6SS production is not following the same Rsm-dependent regulation than biofilm. This Rsm-independent regulation of T6SS has to be confirmed. As AlgU is exerting a negative effect on HSI-I genes (Tart *et al.*, 2005), this could explain at least the KK14 defective T6SS production.

KK72 strain exhibits a deregulated T3SS production and secretion (effective in non-induced and induced conditions, Figure 1E) that is coupled to an increased ExsA amount. Beside high levels of free RsmA that positively regulates *exsA* expression [47], additional levels of regulation might be implied, T3SS expression and activity being finely tuned (for review [48,49]). No particular mutation was found specific to this KK72 strain in key regulatory T3SS genes such as *vfr* and *exsC* and *exsE*. Examination of the promoters and genes of the transcriptional activator ExsA and its repressor ExsD was made indicating that ExsD accumulates mutations in KK14 and KK72 as compared to KK1 strain such as P126A and G235V; whether these mutations participate in hyper production of T3SS in KK72 strain remains to be studied. Additionally, the negative T3SS producing KK14 strain exhibits a unique S232R mutation in ExsA, whether it contributes to its DNA binding activity remains to be elucidated. ExsA expression, the master regulator of T3SS gene expression, has been demonstrated to be reduced in *mucA* mutants through either a Vfr-independent mechanism involving the RsmAYZ regulatory system and the TCS AlgZ/AlgR [47] or a Vfr-dependent mechanism [50] possibly on the promoter of the regulatory operon *exsCEBA* [51]. Whether it contributes to absence of T3SS expression in the mucoid KK14 strain has to be elucidated as well.

**Figure 6:**
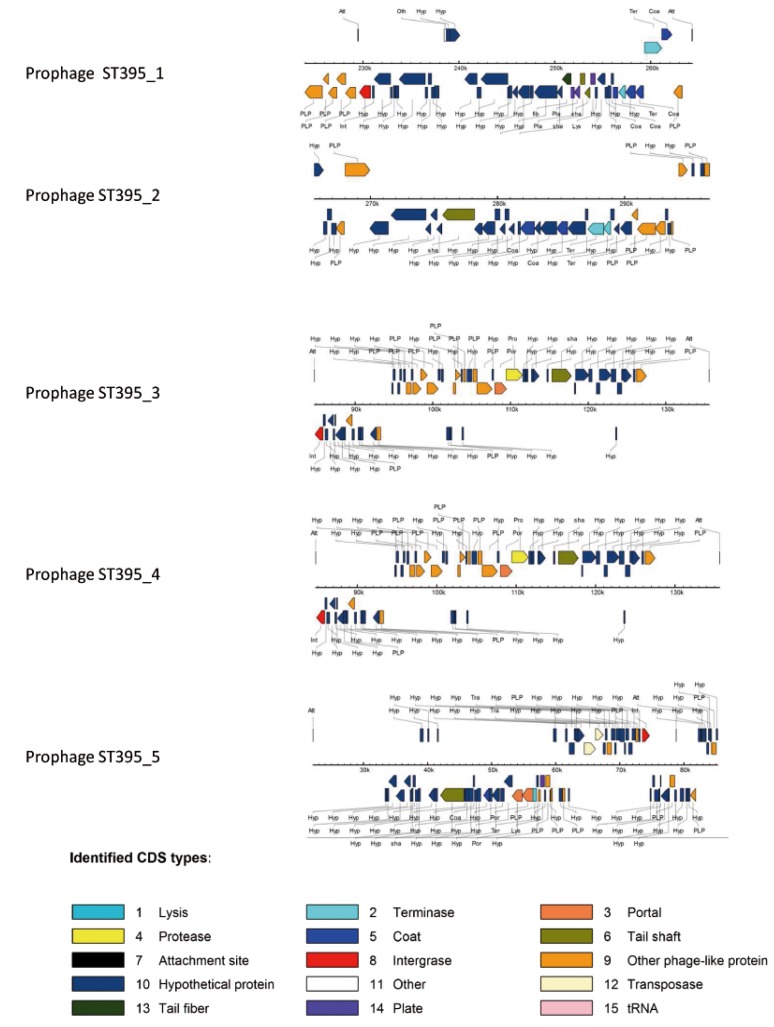
Prophages identified in ST395 strains. The identification has been performed using PHAST software; each arrow represents a gene predicted by PHAST and color codes indicate the corresponding functions of the genes.

#### 2.2.2. ST395 Strain Microevolution and Phenotypic Consequences

**Figure 7:**
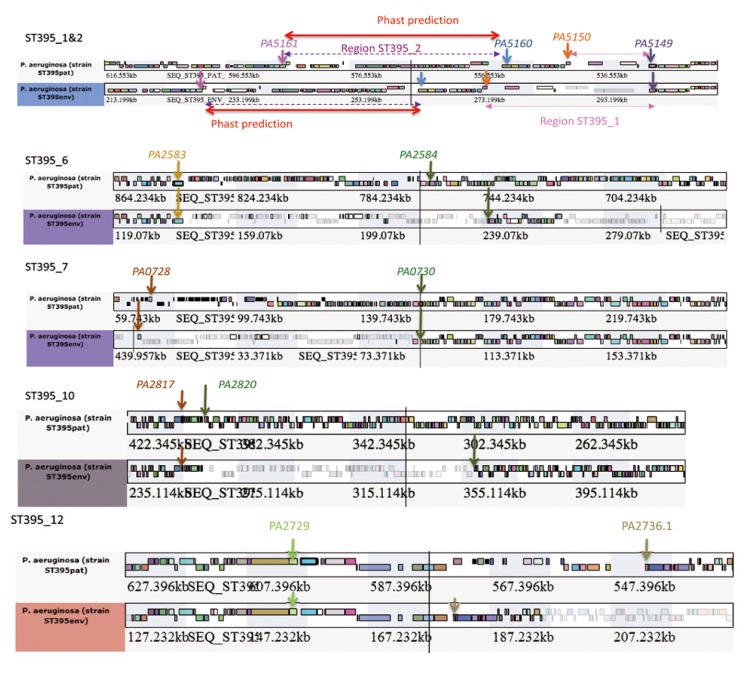
ST395 strain RGPs varying between ST395 strains using Wallgene facility. ST395P was chosen as the reference genome. The same color codes presented for Figure 5 are used.

As proposed previously, an even short-term habitat differentiation can cause major phenotypic diversification driven by single genomic variation events and uptake of phage DNA (Bezuidt, 2013). Rearrangements observed in ST395 strains (Table 4 and Figure 6, Figure 7 and Figure S2) are good illustrations of this feature in a non CF clinical context. 

**Table 4:** Regions of genome plasticity of the sequenced *P. aeruginosa* ST395 strains.

	Location in PAO1 genome	Phagic origin	ST395E	ST395P
Region ST395_1&2	PA5149.1	no	+ (26.483 kb)	+ (12.871 kb)
Region ST395_1&2 Prophage ST395_1	PA5160.1	yes ΦCTX	+ (40.4 kb)	+ (22.7 kb)
Region ST395_3 Prophage ST395_2	PA2603.1	yes Φ297	+ (31 kb)	-
Region ST395_4 Prophage ST395_3	PA4138-PA4139	yes F10	+ (42,196kb)	?
Region ST395_5 Prophage ST395_4	PA2794-PA2795	yes F116	-	+ (70.9 kb)
Region ST395_6	PA2583.1	no	+ (99.342 kb)	+ (79.699 kb)
Region ST395_7	PA0728 -PA0730	no	+ (91 kb)	+ (104.3 kb)
Region ST395_8	*phnA* -*phnB* genes	no	-	+ (74 kb)
Region ST395_9 Prophage ST395_5	PA3824.1	yes B3	-	+ (63.1 kb)
Region ST395_10	PA2817-PA2820	no	+ (104.5 kb)	+ (7.46 kb)
Region ST395_11	PA0976.1	no	+ PAPI-1 like	+ PAPI-1 like
Region ST395_12	PA2730-PA2736.1	no	+ (25.6 kb)	+ (57.25 kb)
Region ST395_13	PA4231-PA4232	no	+ (19.6 kb)	+ (19.6 kb)
Region ST395_14	PA3835-PA3836	no	+ (PAGI-9) (7.192 kb)	+ (PAGI-9) (7.192 kb)

##### 2.2.2.1. Major Genomic Changes between ST395 Strains

The ST395_1 region was identified in place of the tRNA-Phe PA5149.1 of PAO1 strain, the location of the previously described RGP62 [25]. However, none of the genes of ST395 strains display homologies with those of PACS2 strain. This region varies in size from 26,483 bp in ST395E and 12,871 bp in ST395P (Figure 7), respectively due to the absence of the last four genes in ST395P.

The ST395_2 region of 40.4 kb in ST395 strains (prophage ST395_1 corresponding to 49 ORF, (Figure 6 and Figure 7), with a GC% of 63.7 and 65.2 for ST395E and ST395P, respectively) was identified in both ST395 strains and present in between PA5160 and PA5161 (*rmlD*) of PAO1 strain in place of the tRNA-Thr PA5160.1.

The ST395_3 region of 50 kb (prophage ST395_2 corresponding to 68 ORF with a GC% of 62.7) was specifically identified in ST395E strain in place of the tRNA-Ser PA2603.1. PHAST predicts an incomplete prophage which displays homology with the phage Φ297 (Figure 6). This region ST395_3 is probably incomplete since present at the ends of two contigs.

The ST395_4 region of 50.8kb (prophage ST395_3 corresponding to 60 ORF with a GC% of 62.8) was identified ST395E strain in a region which was not previously identified as a RGP [25]. Its existence in ST395P could not be ascertained since both bording genes were present at the ends of two contigs. This prophage (Figure 6) is homologous to prophage 2 identified in LESB58 strain of which 32 genes are homologous to the sequenced bacteriophage F10 [52].

The ST395_5 region (prophage ST395_4 corresponding to 70 ORF with a GC% of 63) of 70.9 kb was specifically identified in ST395P strain between PA2794 and PA2795 genes of PAO1 strain. This region is an intact prophage which displays homology with the bacteriophage F116 (Figure 6).

The region ST395_6 was identified in both ST395 strains in place of the tRNA-Gly PA2583.1 of PAO1 strain with a respective size of 99,342 and 79,699 bp in ST395E and ST395P strains (Figure 7). In ST395E it represents part of RGP29 of strain 2192 lacking the Dit Island genes encoding proteins of abietane diterpenoids metabolism [25], while in ST395P, this region is organized as the RGP27 of PACS2 [25]. Thus, whereas both ST395 display additional regions in this genome location, it appears that these two regions are not well conserved between the two ST395 strains, resequencing this region with NGS long reads technology could thus be decisive.

The region ST395_7 inserted in between PA0728 and PA0730 from PAO1 strain, described as the RGP5 [25], is in ST395E an homologous region of 91kb (PSTAB_1168-PSTAB_1251) identified in *Pseudomonas stutzeri,* strain ATCC 17588 (LMG 11199) [53]. In ST395P strain, bording genes of this DNA region of 104.3kb are homologous to PA7_5324 and PA7_5328 and the inserted region derives from the *P. aeruginosa* NCGM2.S1 strain and thus differs widely as compared to the corresponding region in ST395E strain (Figure 7). For example, ST395P strain has an *int1*-like gene (99.9% of identity), a trace of an integron but no complete cassette gene could be identified. There is a questionable prophage (11 ORF) related to the Pf1 prophage in this region for ST395P but no homologous phage was found using PHAST for ST395E.

The region ST395_8 of 67.15kb was specifically identified in ST395P strain inserted in between *phnA* and *phnB* genes of PAO1 strain. This region is part of the RGP29 (PA2G_02071-PA2G_02148) identified in strain 2192 of *P. aeruginosa* [25].

The region ST395_9 of 63.1 kb was also specifically identified in ST395P strain (prophage ST395_5 corresponding to 85 ORF with a GC% of 63.2) inserted in place of the tRNA-Leu (PA3824.1) of PAO1 strain, however the other bording gene is unavailable due to the end of the corresponding contig (Figure 6). The region exhibits high homologies with the region of 39016 strain of *P. aeruginosa* (PA39016_000840119-PA39016_000840070) derived from Phage pseudoB3. 

Additionally, these ST395 strains were characterized by several other major genomic rearrangements. For example, the region between PA2817 and PA2820 in PAO1 strain containing the gene encoding the aminoglycoside response regulator (Arr) [27] is varying in between the two ST395 strains (ST395_10) and compared to PAO1 strain (Figure 7). In ST395E, this region is 104 kb long whereas in S395P this region is only 7.46 kb long, suggesting that most of this region was lost in ST395P. Genes present in this region in ST395P strain are also present in ST395E strain. In ST395E, additional region resembles the RGP29 inserted locus of PAGC2 strain but lacking the Dit Island genes encoding proteins of abietane diterpenoids metabolism [25].

The ICE PAPI-1 identified in PA14 strain was found in both strains (ST395_11) but instead of being inserted in the PA4514.1-4541.3 tRNA region as in PA14 strain [54], this ICE is inserted in place of the tRNA-Lys PA0976.1 in both ST395 strains, the place where the ICE PAPI-2 is inserted in PA14, this latter region being identical to PAO1. Whether this ICE PAPI-1 is complete requires extended PCR since genes of the end of this PAPI-1-like element are present at the ends of sequenced contigs in both strains.

The region ST395_12 is located in place of PA2730-2736.1 of PAO1 strain. This region of 25.6 kb and 57.25kb in ST395E and ST395P (Figure 7), respectively is containing genes common to both strains but STP395P contains additional genes encoding an integrase, a protein involved in DNA repair (RadC like) and a protein homologous of ThiJ from *E. coli* belonging to the DJ-1 superfamily [55].

The region ST395_13 present in both ST395 strains of 19.6 kb is located in between PA4231 (*pchA*) gene and PA4232 (*ssb*) gene of PAO1. It contains a majority of genes encoding integrase family proteins with high homologies with those identified in *P. putida* GB-1 strain.

Glycosylation locus involved in A-band and B-band lipopolysaccharide synthesis [56] is absent in draft genomes of ST395 strains.

All phage regions were confirmed by mapping reads and therefore we could confirm that when stated these regions are missing.

##### 2.2.2.2. Minor Genomic Changes between ST395 Strains

More discrete remodeling events were noticed in ST395 strains. For example, the region upstream the *cupA* locus, which contains the *cgrABC* genes controlling *cupA* gene expression [57,58], exhibits in ST395P a probable frameshift in *cgrA* gene. Whether this disturbs *cgr*-dependent *cupA* regulation in this strain requires functional studies.

The PA2152 gene encoding a protein with maltose alpha-D-glucosyltransferase activity probably involved in trehalose biosynthesis in PAO1 strain [59] exhibits premature stop codons in both ST395 strains, possibly suggesting that this pathway is altered in ST395 strains.

ST395 strains possess additional genes such as a gene encoding an asparagine synthetase recovered only in LESB58 and M18 *P. aeruginosa* strains in between *rfaE* [60] and PA4995 genes of PAO1 strains, a gene in between PA2790 and P2791 genes of PAO1 recovered in LESB58 and B136-33 *P. aeruginosa* strains whose corresponding putative product contains a right handed beta helix region that shares some similarity with pectate lyases. The PA3164 gene which in PAO1 strain seems to be a pseudogene due to frameshift is probably encoding a functional 3-phosphoshikimate 1-carboxyvinyltransferase prephenate dehydrogenase in both ST395 strains. Other discrete variations were identified in particular in *algP* gene from ST395 strains, the gene which encodes a regulator involved in regulation of mucoidy in *P. aeruginosa* [29]. None of the open reading frame of these strains was matching with the *algP* wild type version, probably highlighting a defect in mucoidy conversion in ST395 strains. Another example comes from the PA2690 gene in PAO1 which encodes a probable transposase which is absent from the ST395 strain genomes. 

Interestingly, ST395 strains possess as B136-33, 2192 and RP73 and PSE9 [61] strains of *P. aeruginosa* an additional gene (ST395_14) present in between PA3835 and PA3836 genes of PAO1 strain. This gene forms the PAGI-9 island of 7.192 kb identified in PSE9 strain [61]. Thus strains ST395 possess the PAGI-9 island formed of the *rhs* gene and could probably contribute to virulence of these strains in acute pneumonia in the context of ventilator-associated pneumonia in ICU [62].

The ST395 strains are thus highly differing in their prophage regions but also major differences are observed between their RGP, suggesting that environmental constraints have highly shaped genome backbones in between the two times of isolation, although intermediate sampling could help in identifying whether changing habitat may have caused these rearrangements.

##### 2.2.2.3. Genetic Changes Related to ST395P Multi-Drug Resistance towards Antibiotics as Compared to ST395E

Genes or regions known to be implicated in resistance to ß-lactams, aminoglycosides and fluoroquinolones were investigated in detail [63,64] (Table 5).

**Table 5:** Genetic events in target genes controlling acquired resistance in ST395 strains.

	ST395E	ST395P
Resistance to ß lactams		
AmpC derepression		
AmpD, AmpDh2, AmpDh3	A134V, wt, R66C	T139M and A134V, wt, R66C
OprD	wt	c703t SNP introducing a premature stop codon
MexAB-OprM overproduction		
MexR	wt	H107P
NalC	G71E	G71E
NalD	wt	wt
ArmR	wt	wt
MexXY-OprM overproduction	See Resistance to aminoglycosides	See Resistance to aminoglycosides
MexCD-OprJ overproduction		
NfxB	wt	wt
Resistance to aminoglycosides		
Aminoglycoside modifying enzymes		
APH(3')-IIb	wt	wt
MexXY-OprM overproduction		
MexZ (*agrZ* mutant)	wt	del nt452-459 of *mexZ* gene
RplA (*agrW1* mutant)	wt	wt
Fmt (*agrW1* mutant)	wt	wt
FolD (*agrW1* mutant)	wt	wt
ArmZ (*agrW1* mutant)	wt	wt
*rplU-rpmA* promoter (*agrW1* mutant)	wt	Insertion of 2g at -186nt before the start codon of the *rplU* gene
ParRS (*agrW2* mutant)	ParR wt, ParS wt	ParR wt, ParS V216A
PA2572-PA2573	wt,R206G Q210K S217A N236D	wt,R206G Q210K S217A N236D
Resistance to fluoroquinolones		
DNA gyrase and topoisomerase		
GyrA	925 aa (PA01 923 aa)	925 aa T83I
ParC	wt	S87L
GyrB	wt	wt
ParE	V200M	V200M
MexAB-OprM overproduction	See Resistance to ß lactams	See Resistance to ß lactams
MexXY-OprM overproduction	See Resistance to aminoglycosides	See Resistance to aminoglycosides
MexCD-OprJ overproduction	See Resistance to ß lactams	See Resistance to ß lactams
MexEF-OprN overproduction	MexT wt, MexS wt	MexT R48C, MexS T19P

*Resistance to ß-lactams.* In *P. aeruginosa,* resistance to ß-lactams may be due to overproduction of intrinsic ß-lactamase AmpC, acquisition of various secondary β-lactamases through horizontal gene transfer, decrease in the outer membrane permeability (loss of porins) [65] and/or overproduction of active efflux systems, mainly MexAB-OprM and MexXY-OprM [66].

**Figure 8:**
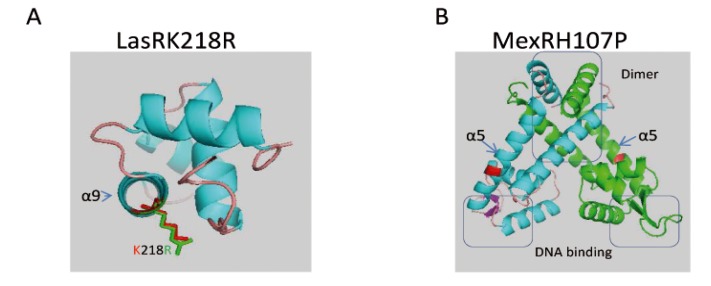
(**A**) Modelization of the C-terminal end of the LasR activator of QS. LasR from KK14 and KK72 differed by an amino-acid substitution K218R (K for KK14, and R for KK72 strains) present in the α9 helix important for recognition of DNA before binding. (**B**) Modelization of the dimer of the MexR variant of the transcriptional repressor of the MexAB-OprM efflux pump in strain ST395P. Each monomer is colored differently (green and blue), both harboring amino acid substitution (in red) in α5 helix (H107P) which is probably not critical for MexR oligomerization or DNA binding.

While no acquired ß-lactamase gene could be detected in ST395P, the strain turned out to harbor a mutation in the *ampD* gene leading to a T139M substitution in amidase AmpD, compared with its wild-type susceptible counterpart ST395E. Inactivation of this enzyme which plays an important role in the recycling of muropeptides during the remodeling of peptidoglycan is a well known cause of AmpC upregulation in *P. aeruginosa* and of pan-resistance to ß-lactams except carbapenems [67]. Interestingly, we found that the decreased susceptibility of ST395P to carbapenems (imipenem and meropenem) was related to a C703T substitution introducing a premature stop codon in *oprD*, the gene which encodes the major uptake pathway of carbapenems in *P. aeruginosa*, namely porin OprD [68]. While no clear evidence was obtained of mutations upregulating efflux pumps in ST395P except for MexXY(OprM) (see below) and a H107P substitution in MexR that could explain the *mexAB-oprM* overexpression, although this substitution located in the α5 helix (Figure 8B) of the protein [69] has not been described as critical for MexR oligomerization or DNA binding [70], both the overproduction of intrinsic ß-lactamase and the loss of porin OprD are sufficient to account for the resistance phenotype displayed by this isolate.

*Resistance to aminoglycosides*. High aminoglycoside resistance in *P. aeruginosa* is due to horizontally acquired aminoglycoside-modifying enzymes (AME). Beyond the AME named APH(3')-IIb, contributing to intrinsic kanamycin resistance, none additional AME encoding gene was identified in the ST395 strains. This result was not surprising by itself in view of the low resistance levels to aminoglycosides (gentamicin, tobramycin and amikacin) displayed by ST395P. Strongly supporting that the efflux system MexXY-OprM is upregulated in this isolate and plays a role in its resistance phenotype, we found a deletion (nt 452–459) in the *mexZ* gene that encodes the TetR-like repressor (MexZ) of operon *mexXY* [71]. Comparatively, ST395E harbored an intact, wild-type gene *mexZ.* Recently, mutational inactivation of MexZ has been reported as the main cause of low-level efflux-based resistance to aminoglycosides in clinical strains of *P. aeruginosa* [72]. It should be noted here that MexXY-OprM is the only pump able to extrude these antibiotics in this organism. Potential other mutations contributing to overexpression of operon *mexXY* in ST395P have been revealed by genome sequencing, the role of which remains to be confirmed (Table 5).

*Resistance to fluoroquinolones.* Accounting for the elevated resistance of ST395P to fluoroquinolones, a T83I mutation could be identified in the QRDR (Quinolone Resistance Determining Region) of the A subunit (GyrA) of the main target enzyme DNA gyrase [73]. Another prevalent mutation (S87L) responsible for high fluoroquinolone resistance in *P. aeruginosa* was characterized in the C subunit (ParC) of the secondary target enzyme, topoisomerase IV. Beside these mutations that strongly affect the interaction of fluoroquinolones with their cellular targets, upregulation of the MeXY(OprM) system was also expected to contribute to some extend to the resistance phenotype of ST395P to these agents.

In final, our whole genome sequencing experiments revealed that ST395P has become multiresistant through a series of mutations activating intrinsic mechanisms that involve drug hydrolysis (ß-lactamase AmpC), membrane impermeability (loss of porin OprD), and drug efflux (overproduction of MexXY(OprM)). This evolution from the environmental isolate ST395E was not dependent upon lateral gene transfer (summarized in Table 5).

## 3. Experimental Section

### 3.1. Origin of Strains

The CF KK strains were sequentially isolated in a CF patient followed at the CF clinic Hannover who died after the isolation of isolate KK72. The ICU ST395 strains were isolated at the Besançon Hospital, ST395E in a sink of an ICU department and ST395P has been isolated in a patient hospitalized in the same ICU unit and under ventilated assistance.

### 3.2. Genome Sequencing and Bioinformatics

Genomic DNA was sequenced on Illumina Hiseq 2000 system (Beijing Genomics Institute, BGI, China). Mean genome coverage varied between 121 to 140 X. The paired-end reads were assembled *de novo* using SOAPdenovo 1.05. The resulting number of contigs varied between 55 to 125 with an average length from 70,000 to 127,000 bases. Genome sizes and sequencing data are indicated for each strain in Table 1 and Table 2.

Annotation of the assembled contig sequences was performed with the Genostar Suite software. The Genostar Suite, together with the reference MicroB database, is an integrated bioinformatics interactive software application dedicated to microbial genome analysis and comparison. MicroB integrates and updates data from several databases, *i.e.*, NCBI Reference Sequence, UniProtKB, ENZYME, Gene Ontology and KEGG. GenoAnnot module was used to annotate the 5 genomes. A variant of the PRIAM algorithm [74] was used to predict the enzymatic activities of the proteins. The five annotated genomes were integrated in MicroB database. The genomic comparative analyses were performed using Genostar Suite and WallGene and datasets from the present study are available in a free access [24].

WallGene is an interactive web application developed by Genostar (Montbonnot, France) in collaboration with the Pasteur Institute (Paris, France) [75], to facilitate large-scale comparative analysis by computing homologies and visualizing syntenies between a set of genomes, The WallGene visualization tools include several types of views, each with a different purpose from a biological perspective. Six views are currently available: (1) the Wall designed to explore homologies and syntenies in a linear way by focusing on genes of interest; (2) the Line Plot and Dot Plot tools allowing comparison 2-by-2 of the general organization of assembled genomes, and explore some biological events such as conservation, inversion, and duplication; (3) the Core Genome exploring common and specific genes of selected genomes; (4) the Circular View showing the overall organization and homologies of organisms in a set of circular graphs; (5) the Gene List view to retrieve genes and homologies according to gene name or function in tabular form. A user guide is available on the web page [24].

Datasets can be calculated with as reference genome any assembled genome of *P. aeruginosa*, or with a reference genome of choice that could be a neo-sequenced and annotated genome to compare directly with clonal derivatives. In the present example, we did both (PAO1 *versus* KK strains and KK1 *versus* KK14 and KK72, and PAO1 *versus* ST395 strains and ST395P *versus* ST395E) using the same parameters (see comparison between Figure 5 and Figure S1 on one hand and Figure 7 and Figure S2 on the other hand). Unidirectional blastP best hits were computed using the following parameters: 80% coverage, 40% identity and 10^−5^ e-value.

PHAST [76] was used to identify, annotate and graphically display prophage sequences within bacterial genomes. Additionally, any absent phage region from a given genome was further checked to be absent using further mapping of crude reads of this genome against the assembled genomes of strains containing it.

### 3.3. Phenotypic Studies

#### 3.3.1. Antibiograms

MHA plates were inoculated with calibrated suspensions of ST395 strains as recommended by the CLSI. 

#### 3.3.2. Motilities

Swarming and twitching behaviors were determined for strains grown on M8 medium supplemented with 0.5% agar for 48 h at 30 °C and on LB medium supplemented with 1.5% agar for 48 h at 37 °C, respectively [77]. All plates were inoculated with bacteria from overnight cultures on LB agar using sterile toothpicks. 

#### 3.3.3. Biofilm

Subcultures of overnight LB cultures were prepared in minimal growth medium M63 prepared at an initial optical density (OD) at 600 nm of 0.10 and inoculated in 96 well microplates in six replicates per strain or in glass tubes. Plates or glass tubes were incubated 24 h at 30 °C. Bacterial biofilm formation was evaluated by crystal violet staining, extraction by ethanol treatment and sonication and measurement of OD at 570 nm with a TECAN device.

#### 3.3.4. Transcriptional Activities

The miniCTX-*rsmY-lacZ* and miniCTX-*rsmZ-lacZ* vectors [41] were introduced in the different *P. aeruginosa* strains and site specific recombination at the *attB* site generated chromosomal *rsmY-lacZ*, *rsmZ-lacZ* fusions. The FRT cassette-excision step was performed, resulting in the generation of strains without tetracycline resistance.

#### 3.3.5. T2SS

Proteolytic activity mainly due to T2SS substrates in *P. aeruginosa* ([78] was tested on TSA plates supplemented with 1.5% milk and after 48 h at 37 °C. 9 spots were done for each strain, proteolytic halo was measured and mean and standard deviation were calculated and submitted to appropriate t-test comparison.

#### 3.3.6. T3SS

T3SS-dependent cytotoxicity was evaluated on J774 cells by measuring LDH release using Cytotoxicity Detection kit (Roch) after 1, 2 and 3 h of contact with bacteria at a MOI of 10 [79]. Induction of T3SS *in vitro* was obtained by adding 5 mM EGTA and 20 mM MgCl_2_ to bacterial cultures at OD_600nm_ of 0.1. When the cells reached OD_600nm_ 1.0, supernatants were collected and analyzed by immunoblot using anti-PcrV, anti-PopB and anti-ExoS antibodies. Cells were 5-fold concentrated and analysed for PcrV, PopB, ExoS synthesis (WC) and secretion (SN). Cytosolic fractions from 50-fold concentrated cells (Cytos) were also subjected to immunoblot analysis with anti-ExsA and anti-ExsD polyclonal antibodies [79,80].

#### 3.3.7. T6SS

Production and secretion of the Hcp1 of the HSI-1 T6SS were assessed as described [81] with cells grown up to OD_600nm_ of 2.0. Extracellular proteins and whole cell lysates were 50-fold and 5-fold concentrated, respectively. 5 µL of each samples were subjected to SDS-PAGE and immunoblotted with polyclonal antibodies anti-Hcp1 [81].

#### 3.3.8. Modelling

Modelling of LasRK218R and of MexRH107P was performed using PyMol.

## 4. Conclusions

Through this study, we identified common genomic features of *P. aeruginosa* strains from two different medical contexts. Among these, it is important to note the key role of prophages in shaping *P. aeruginosa* genomes, the Arr region which is systematically different in all strains from the one from PAO1 genome (with extra-pieces of DNA ranging from 7,460 to 122,566 bp), and whatever is derived from CF (chronic evolution) and ICU (fast evolution) infections. Of note is the systematic absence in our set of strains of the glycosylation locus involved in A-band and B-band LPS synthesis. Something notably different between CF and ICU strains is that whereas RGP are highly conserved in CF strains, they could have reduced size. In ICU-derived strains we identified RGP present at the same location, which, however, differed from one to another (ST396_6 and ST395_7). This could probably reflect the variability of environmental constraints which is probably much higher in hospital than in a CF lung environment. Furthermore, genome examination of CF strains allowed us to put RGP into three categories which are: (1) RGP which are present in early or mid-term colonization but absent in late clones (regions KK_1 and KK_3); (2) RGP which are continuously present over the period of sampling in KK strains (regions KK_4, KK_5, KK_7, KK_8, KK_9, KK_10, KK_11 and KK12); and (3) RGP which could be considered as “fitness regions”, whose loss or maintenance could improve clonal fitness at one stage of the disease (regions KK_6, the PA1430-1433 region and the case of *mucA* gene). This latter category illustrates the fact that the clones studied here probably represent the ones with the most adequate fitness regarding the CF airway environment at that stage. Several clones with less appropriate fitness could coexist but were not picked up. It has been reported that high phenotypic diversity was apparent in the P. aeruginosa populations from each chronically infected CF patient [82]. Contemporary isolates from a single sputum sample can differ at the SNP, indel, and accessory genome levels and the cross-sectional genomic variation among coeval pairs of *P. aeruginosa* CF isolates can be comparable to the variation previously reported to differentiate between paired longitudinally sampled isolates [9]. Additionally, in a panel of 135 concurrent *P. aeruginosa* isolates from eight different adult CF patients (9 to 20 isolates per patient) for various QS-controlled phenotypes, most patients contained complex mixtures of QS-proficient and -deficient isolates [83]. All these evidences support the fact that there is likely to be a “cloud” of variation and K14 and KK72 that just represent one isolate from amongst the diversity. We thus essentially observed a final reduction of genome size of CF strains along the disease. Regarding ICU strains studied here, as mentioned above regarding the differences observed between their RGP, this could suggest that environmental constraints have highly shaped genome backbones in a very short period (between the two times of isolation), although intermediate sampling could help in identifying whether changing habitat may have caused these rearrangements. We finally observed an increase in their genome size, while switching from a sensitive to a resistant strain to antibiotics.

We thus unraveled diversity of genomes in between clonal derivatives and the potential key role of prophages in shaping bacterial genomes in these two clinical contexts. It is very tempting to speculate that both types of environments (CF or VAP (ventilator-associated pneumonia) in ICU) are selective pressure conditions that result in the changes observed in these *P. aeruginosa* strains. The examination of these genomes at the different levels (contigs, locus, gene) questions the reliability of genotyping methods used to classify strains in a clonal lineage. The availability of genome sequencing facilities would probably help in redefining the clonal notion. 

From our dataset, we would like to go further in depth into the relationship between genetic or genomic events that we observed and phenotypic traits of our strains. This is illustrated for example for KK strains by the Rsm-independent T6SS regulation in KK14 and KK72 strains which can probably be attributed to AlgU in KK14 or to an unknown mechanism in KK72; the role of KK_8 region in antifungal defense in CF airways; the absence of the KK_2 region in CF strains, a region described as essential for increased ability to persist in a chronic pulmonary animal model; the contribution of K218R in LasR in KK72 strain to the absence of proteolytic activity of this strain and the role of G235V mutation in ExsD in deregulated T3SS in KK72 strain or of S232R mutation in ExsA in T3SS defective phenotype of KK14. For ST395 strains, it is of interest to test how the single or multiple punctual mutations identified in AmpD, OprD, MexR, MexZ, *rplU-rpmA* promoter, ParS, ParC, MexT and MexS observed in the ST395P strain act in synergy to shape the resistant phenotype of this strain, because most of these mutations have not been described yet. It would be also very interesting to study whether the RGP ST395_14 region could contribute to the success of this clone in the VAP context. 

Thus, possible sequencing application and easy access to draft genomes would probably contribute towards a virulence and antibiotic resistance survey in clinical contexts in the near future. 

## Supplementary Files

Supplementary File 1
