# Supplementary figures and images for: Pseudomonas aeruginosa Genome Evolution in Patients and under the Hospital Environment
Source: Pathogens. 2014 Apr 10;3(2):309–40. doi: 10.3390/pathogens3020309 (PMC4243448; doi:10.3390/pathogens3020309)

Figure S1

KK\_1

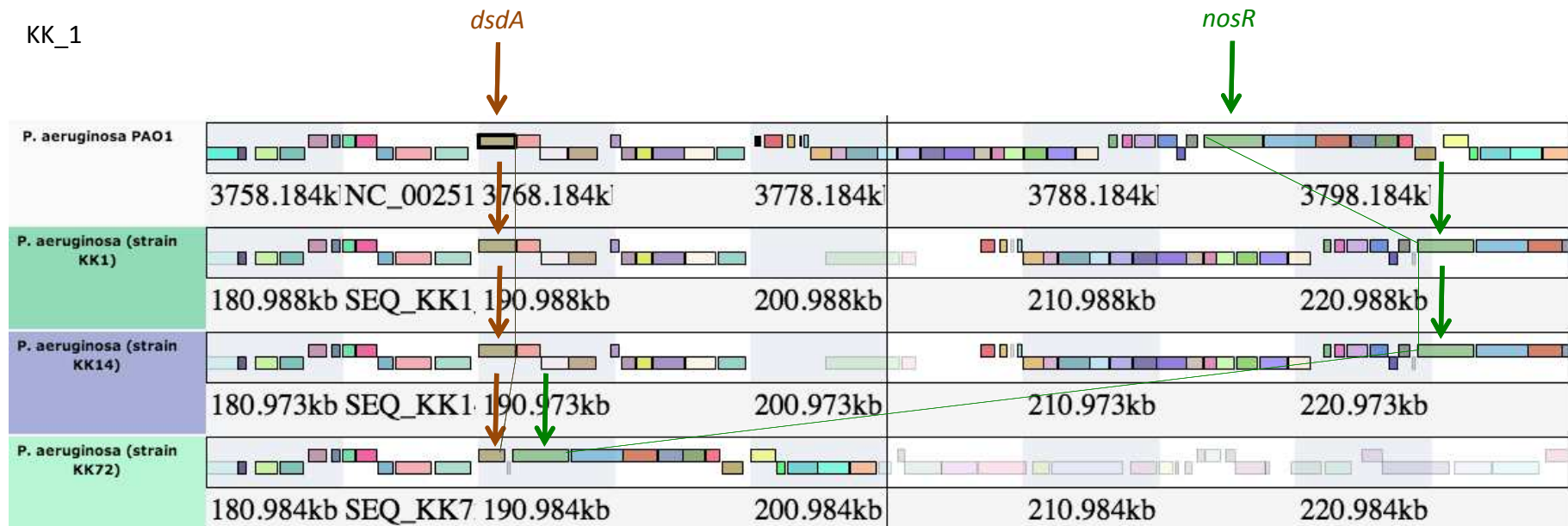

KK\_2

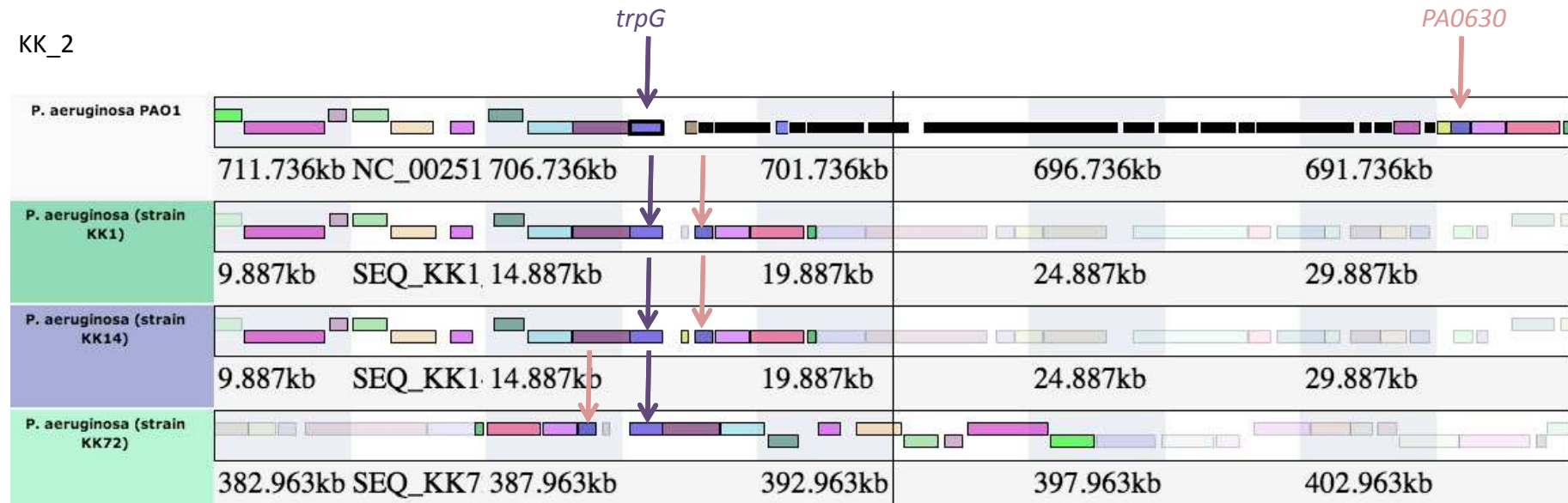

KK\_3

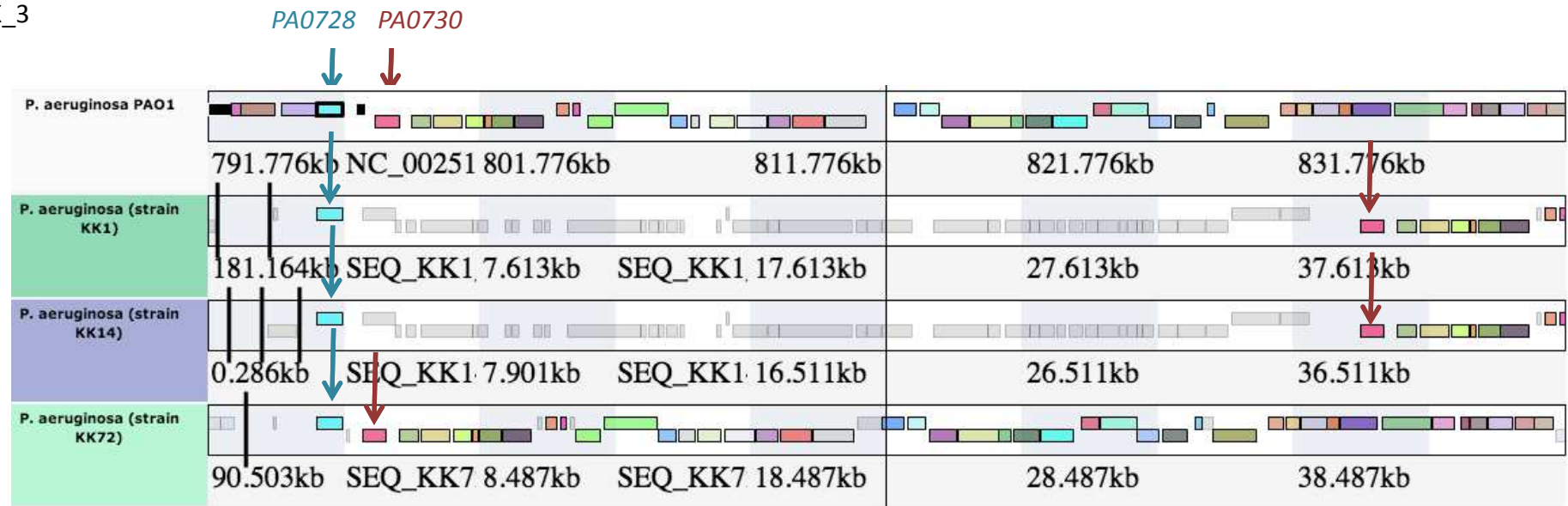

KK\_4

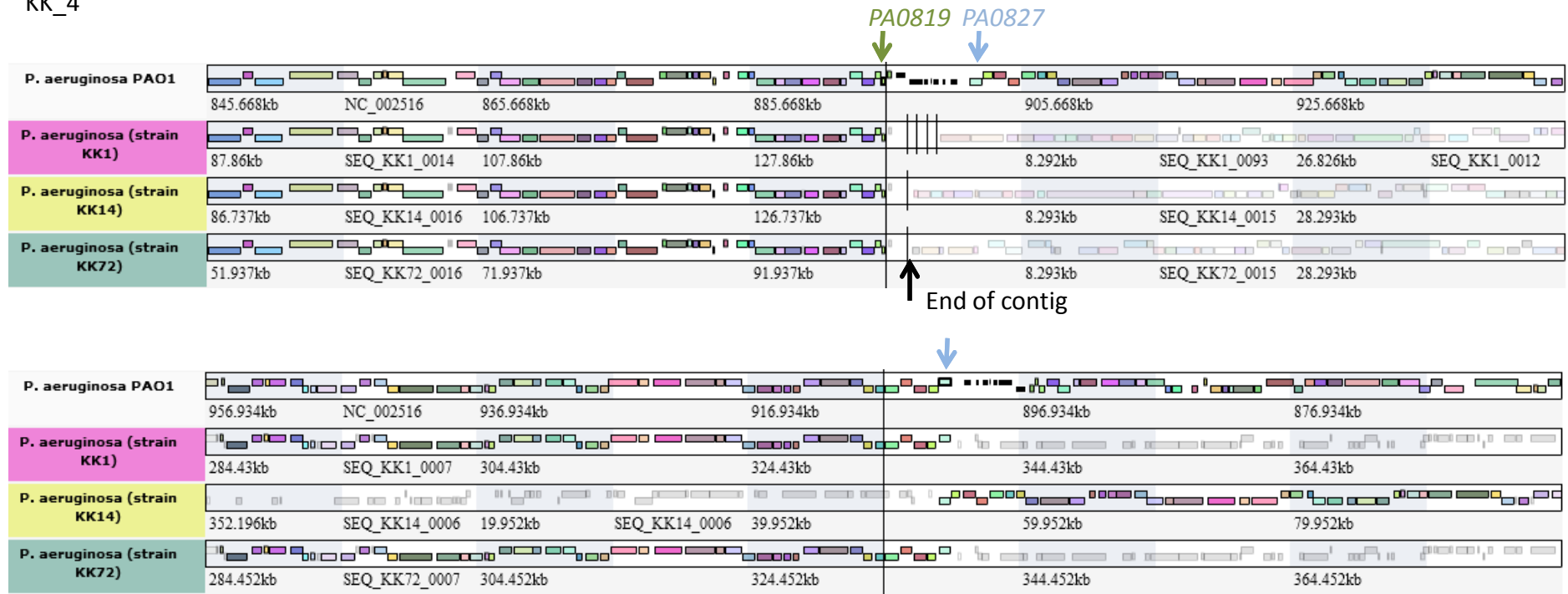

KK\_5

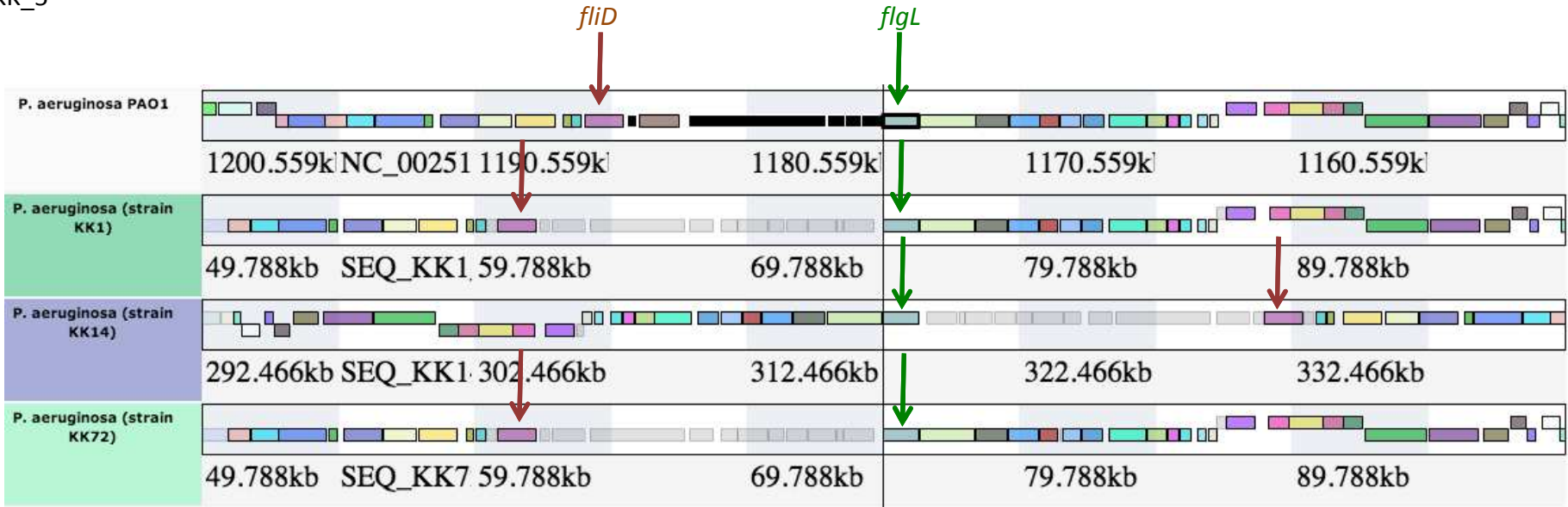

KK\_6

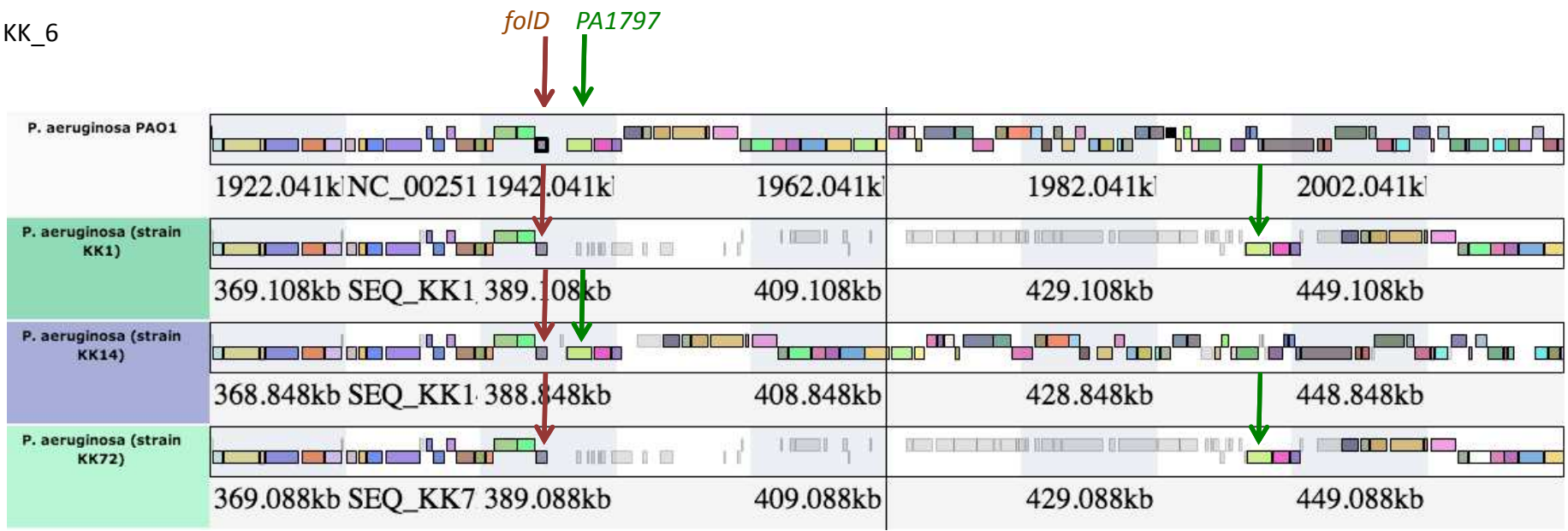

KK\_7

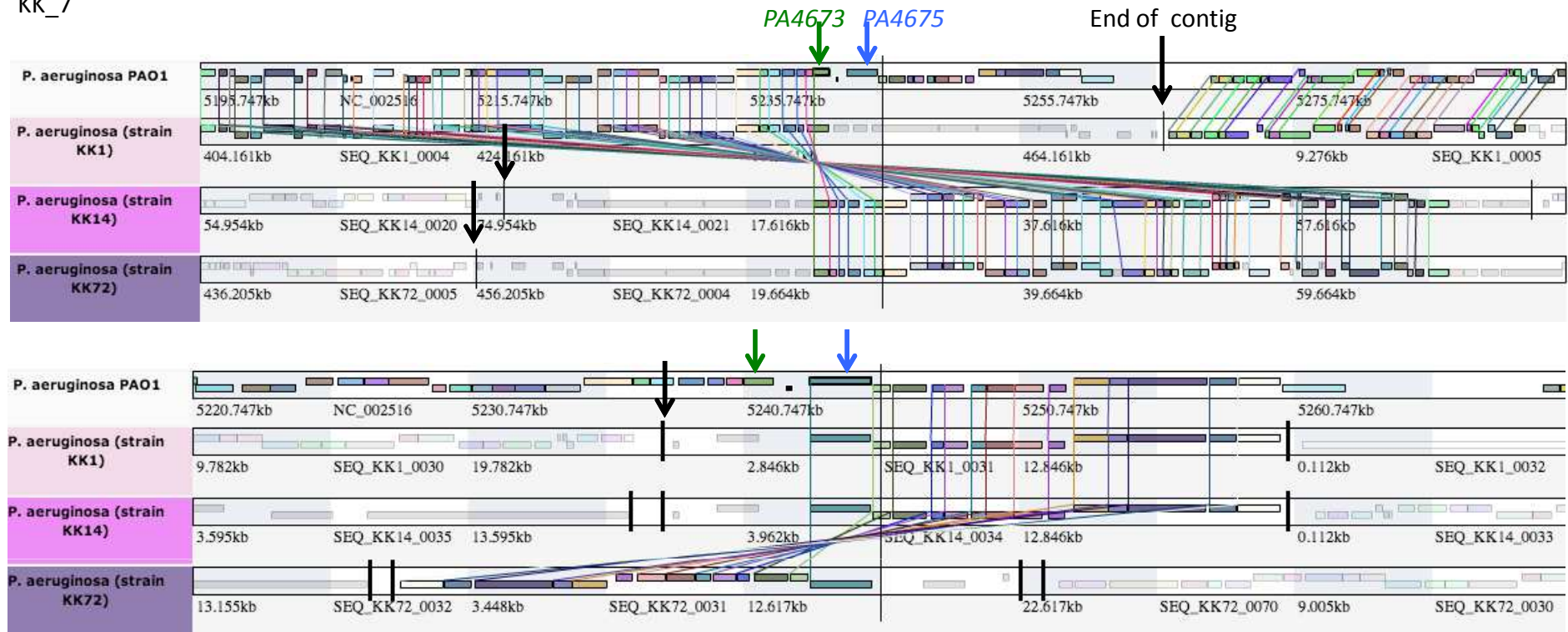

KK\_8

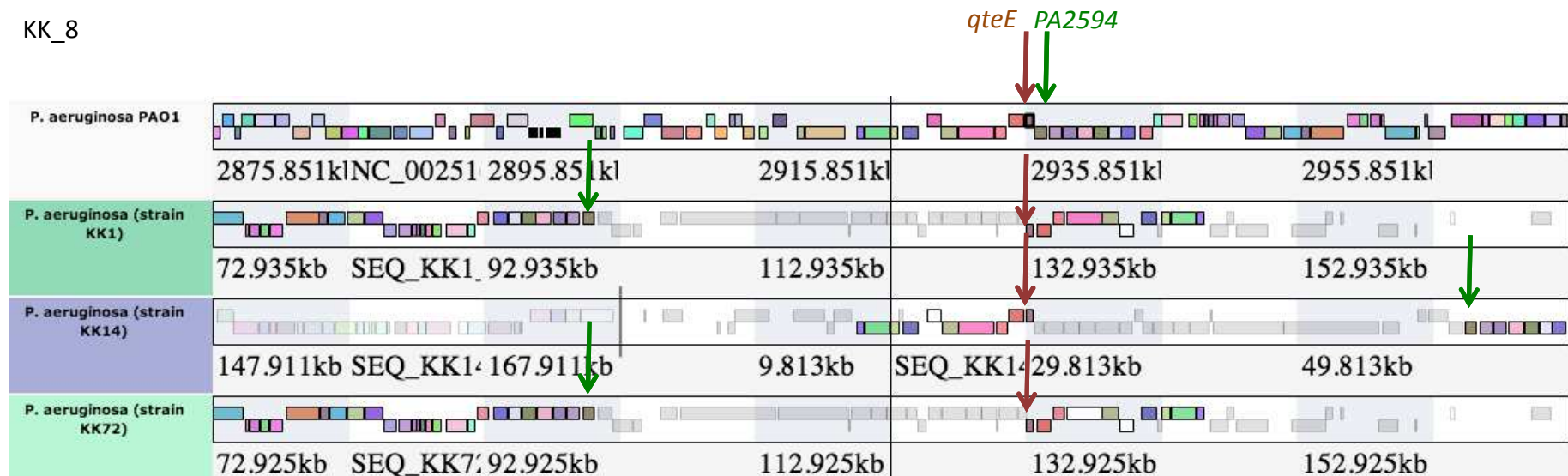

KK\_9

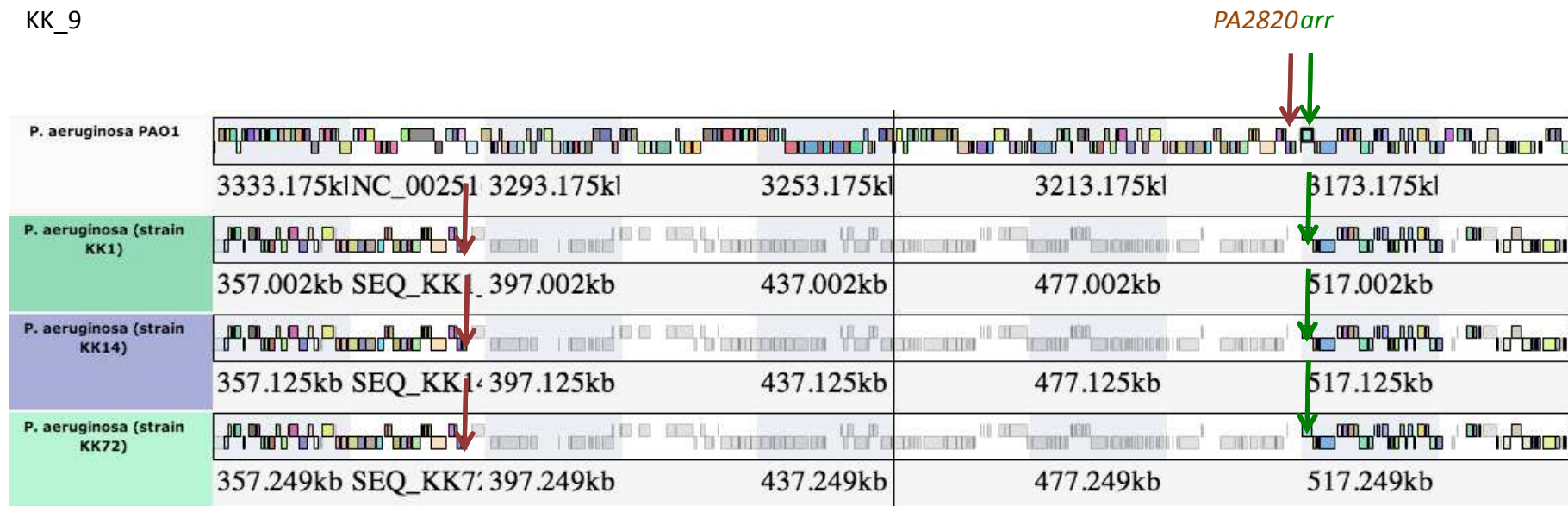

KK\_10

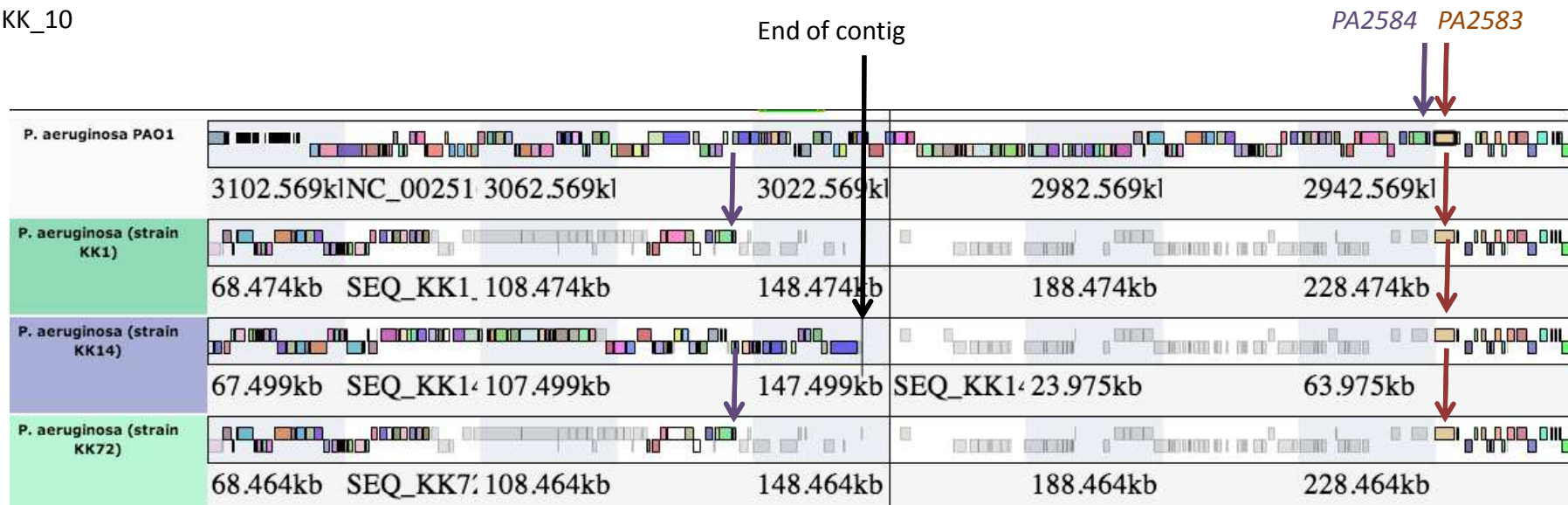

KK\_11

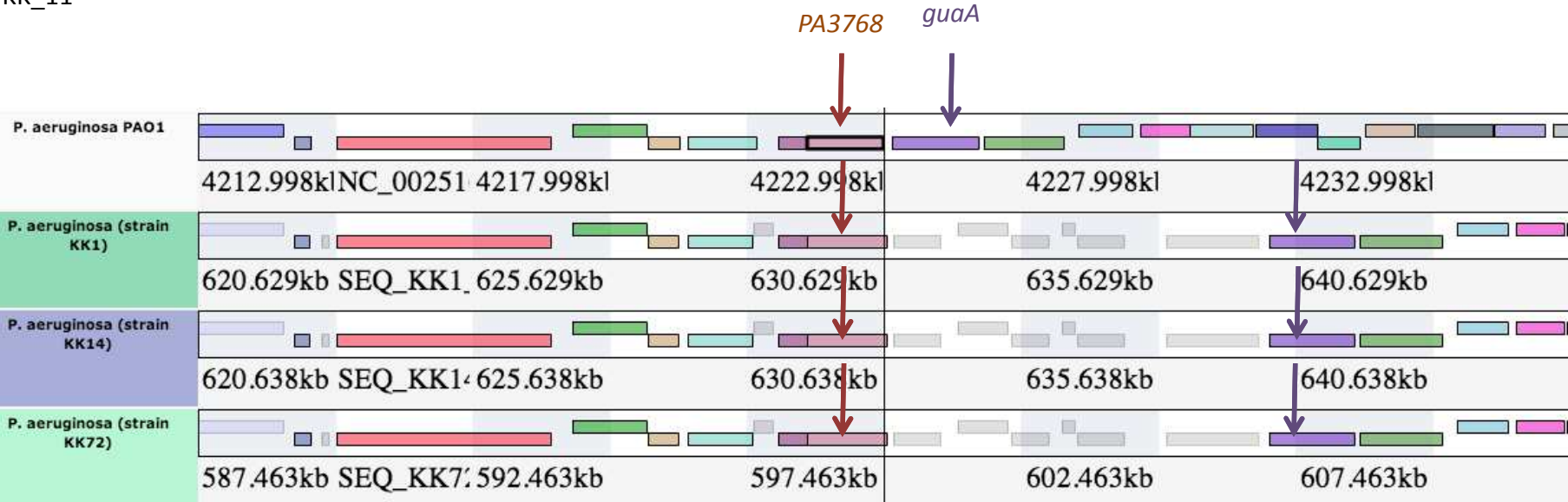

KK\_12

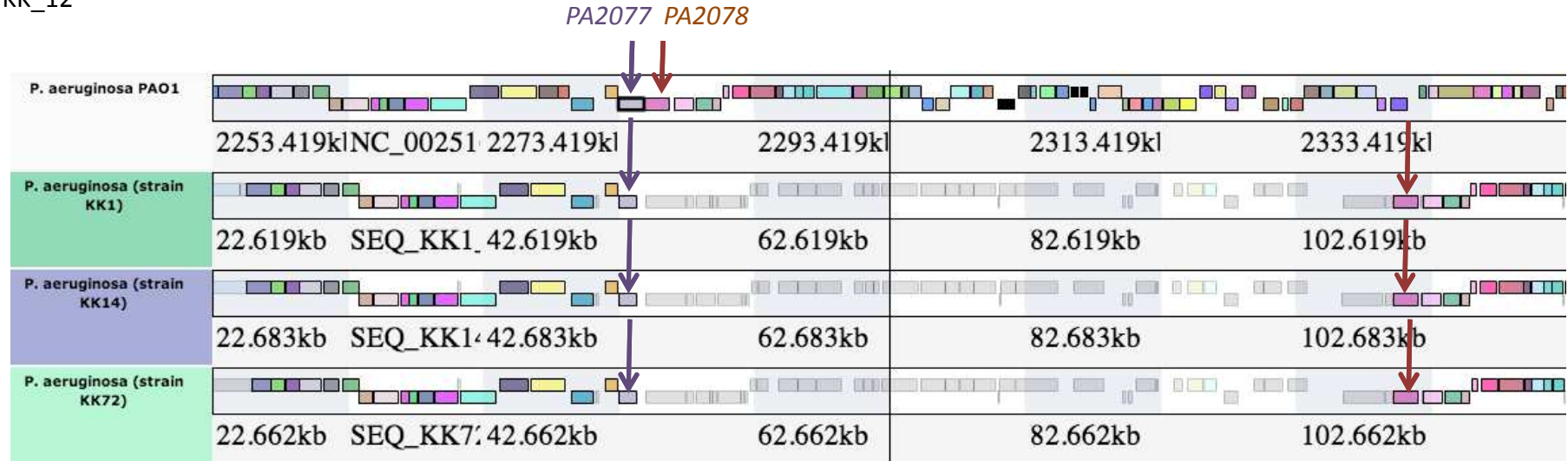

Figure S2

ST395\_1&2

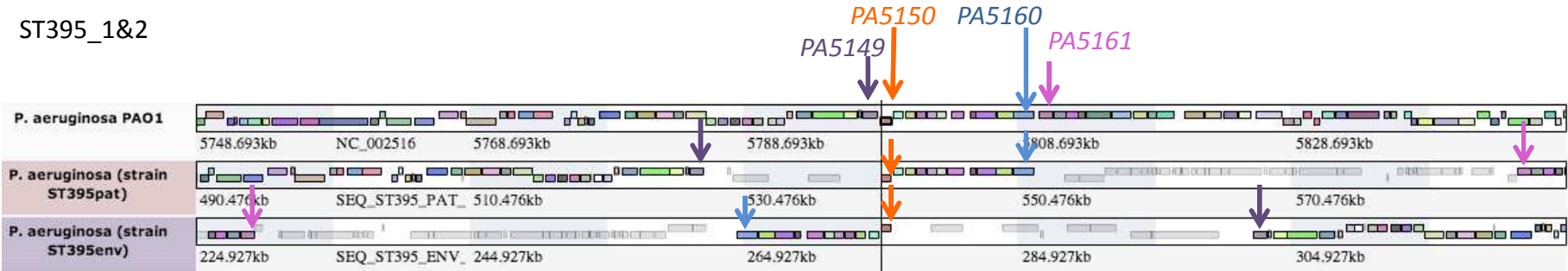

ST395\_3

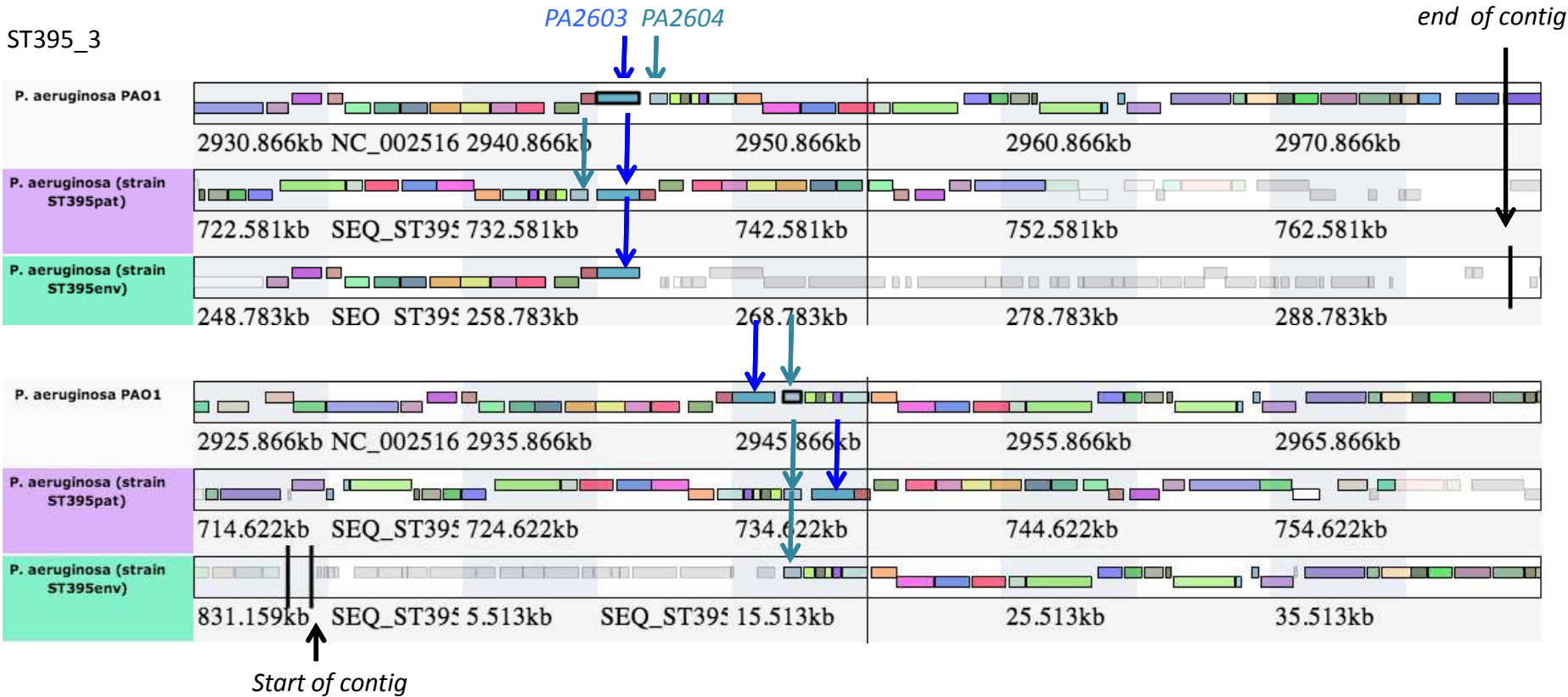

ST395\_4

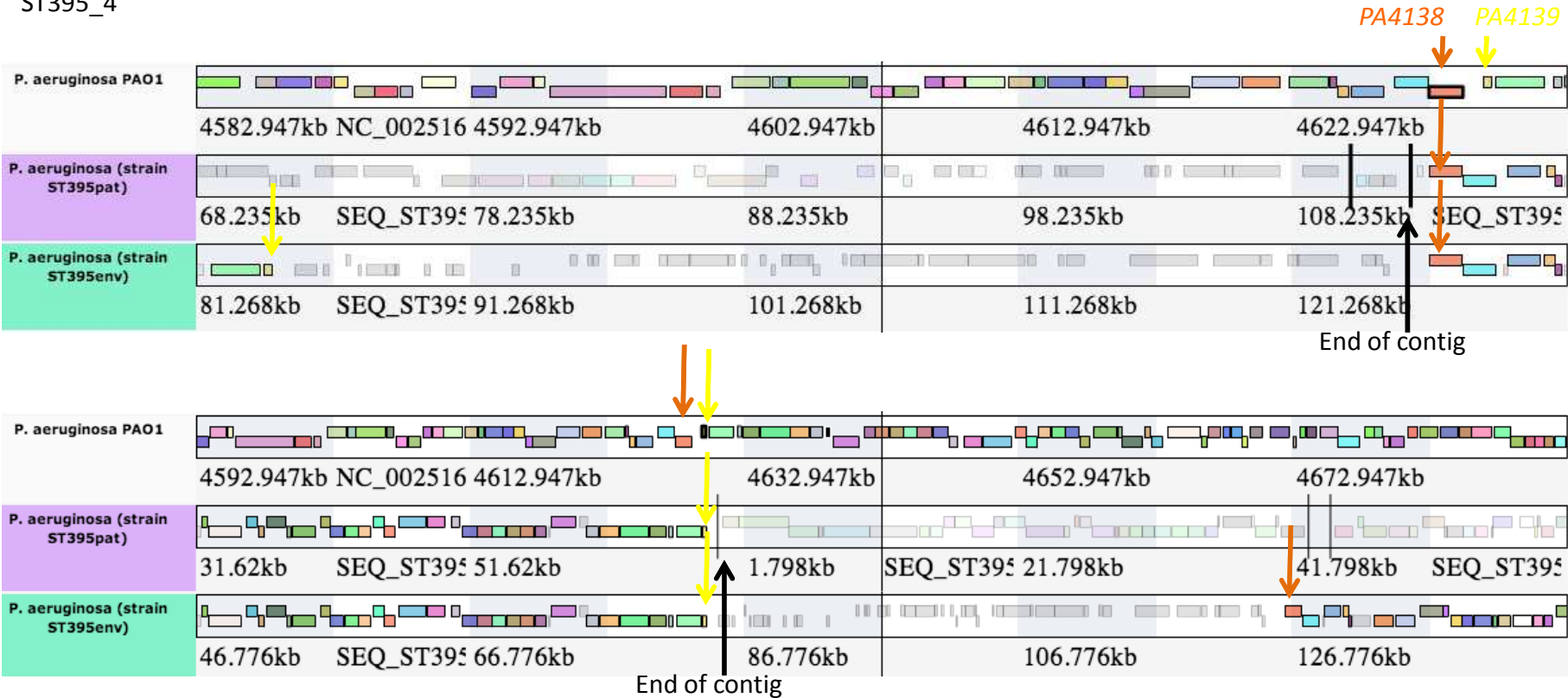

ST395\_5

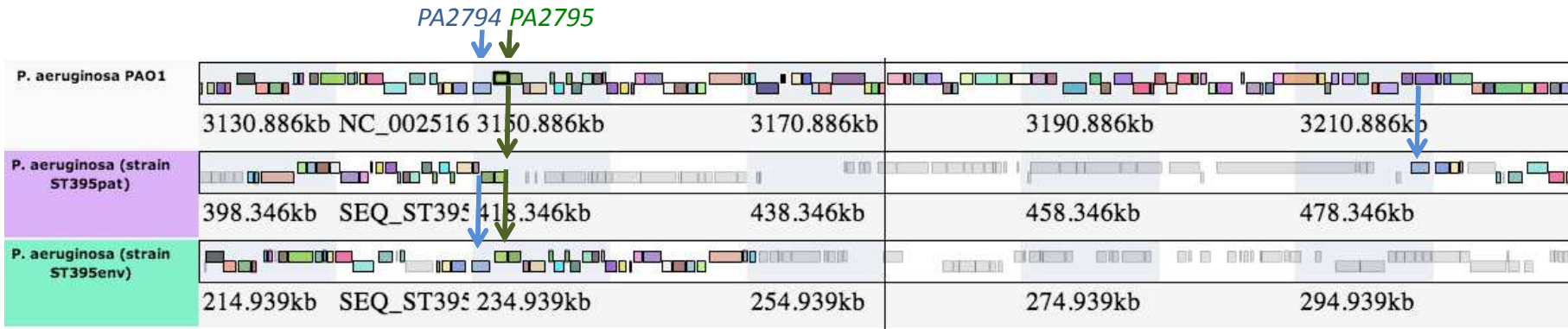

ST395\_6

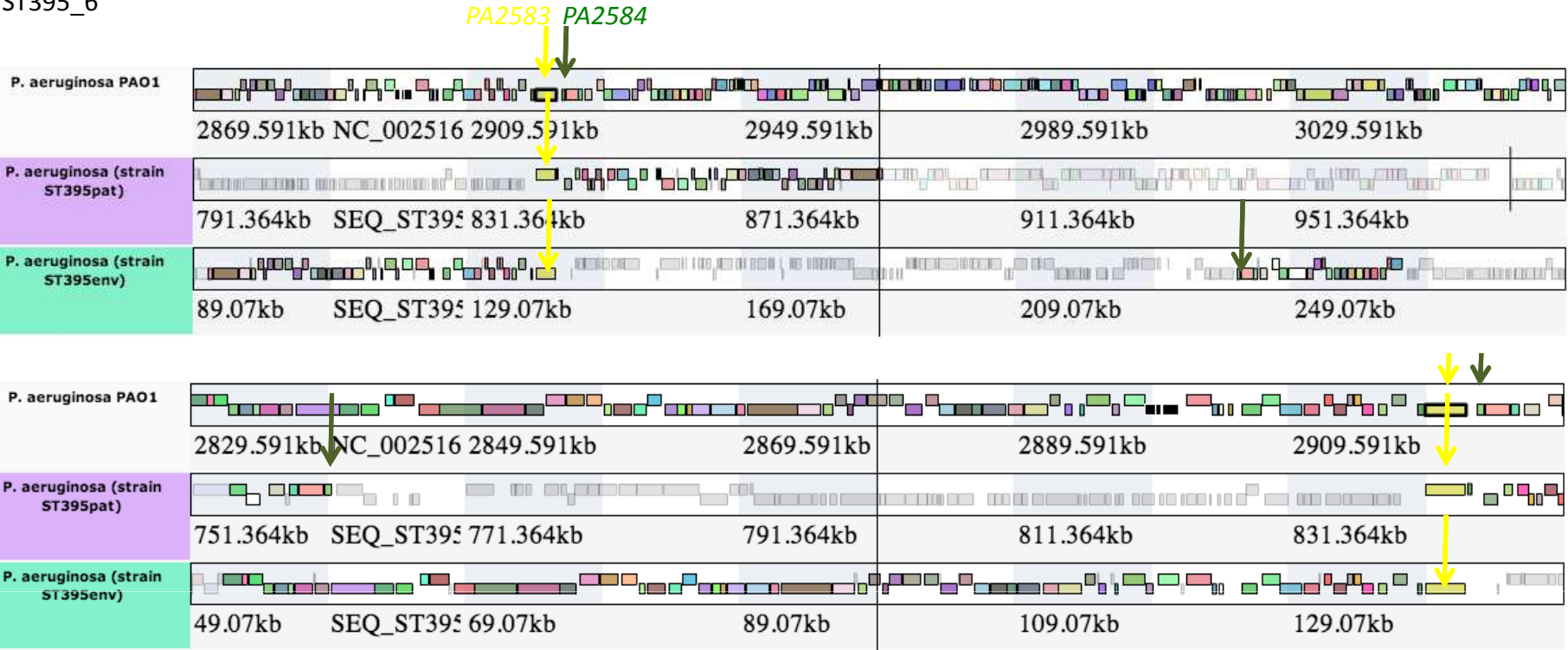

ST395\_7

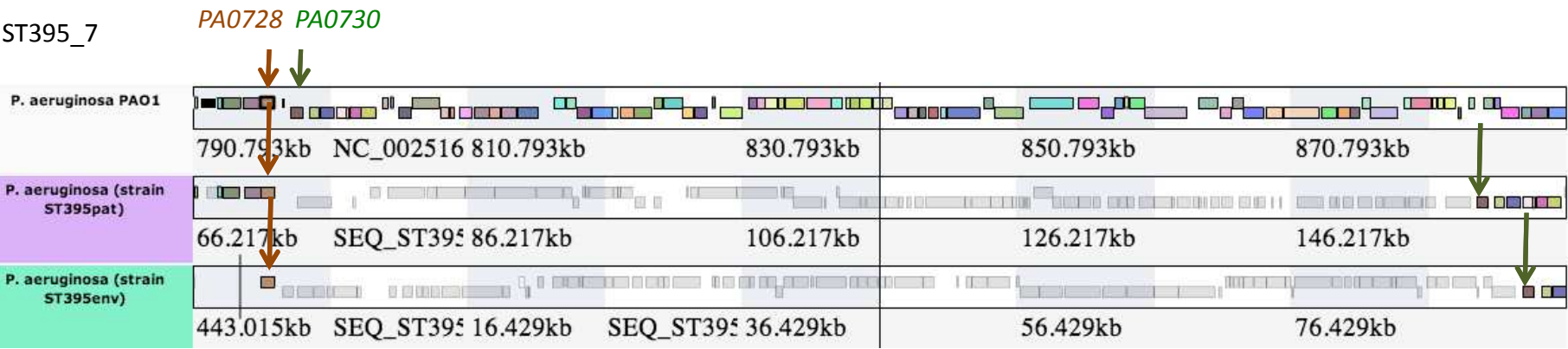

## ST395\_8

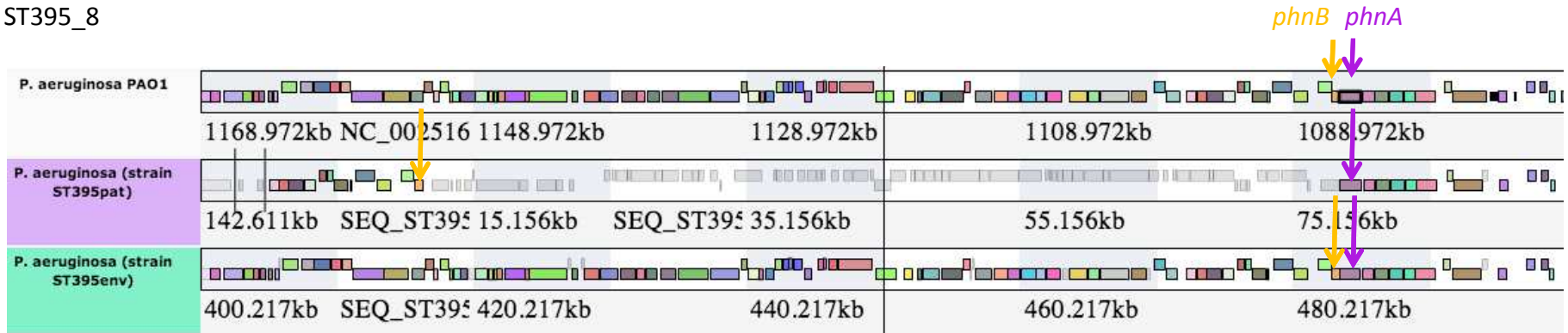

## ST395\_9

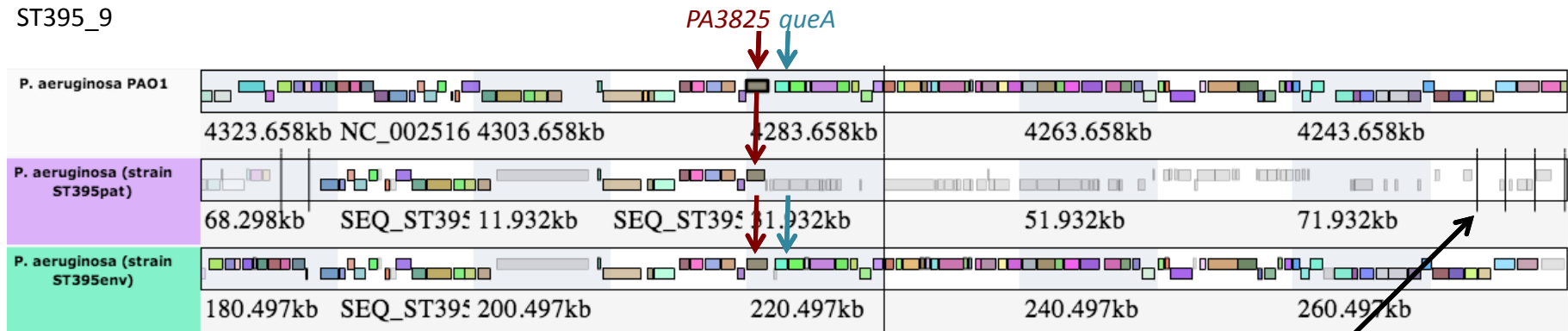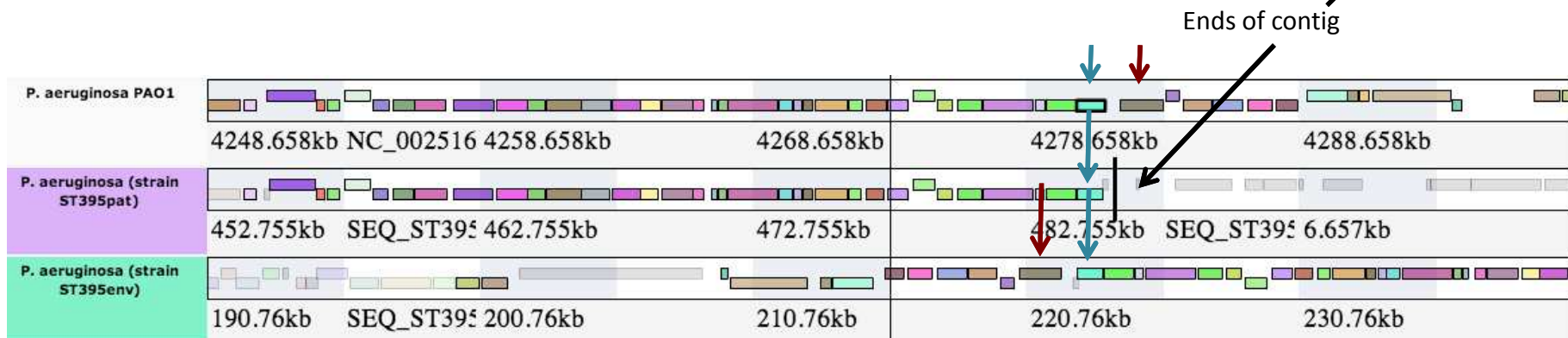

ST395\_10

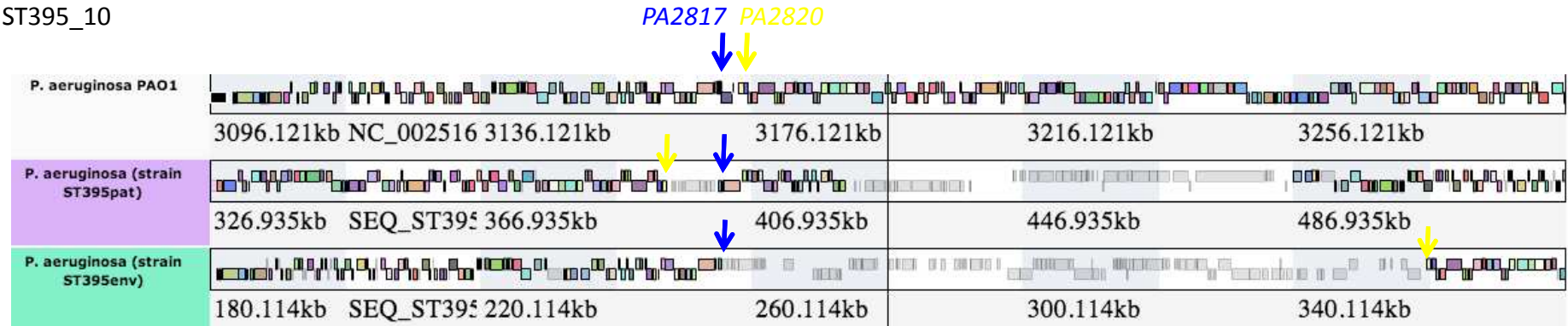

ST395\_11

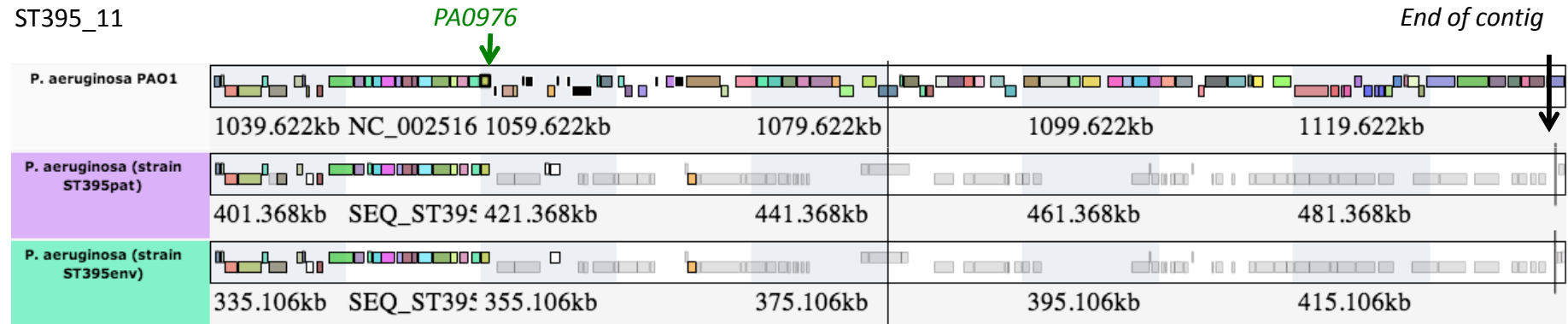

ST395\_13

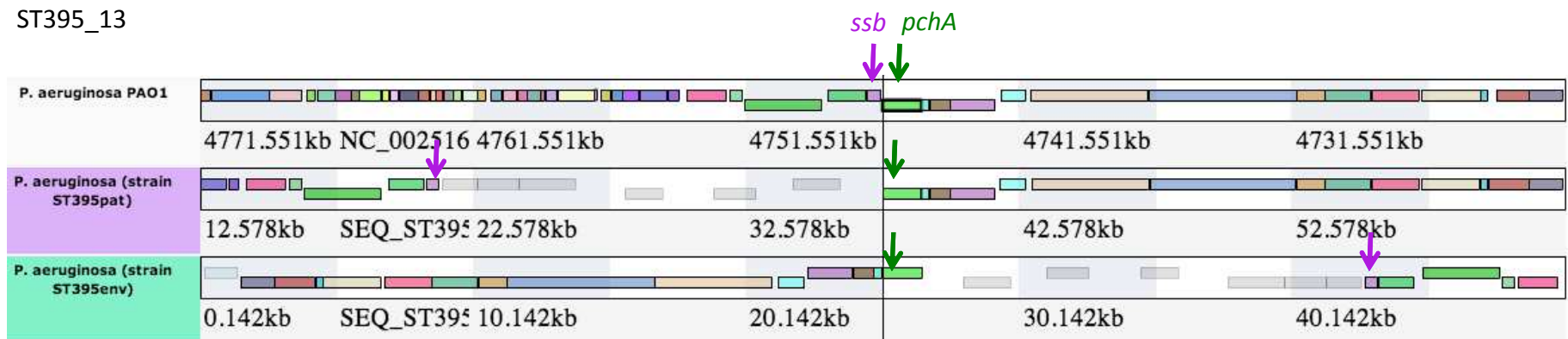

ST395\_12

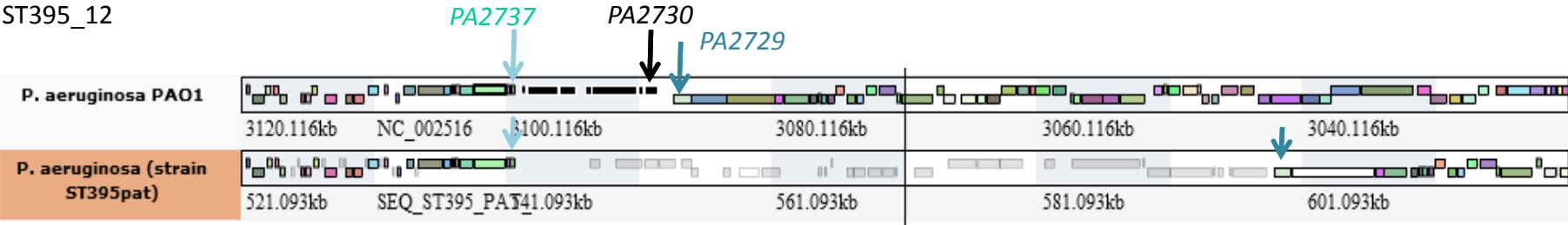

ST395\_13

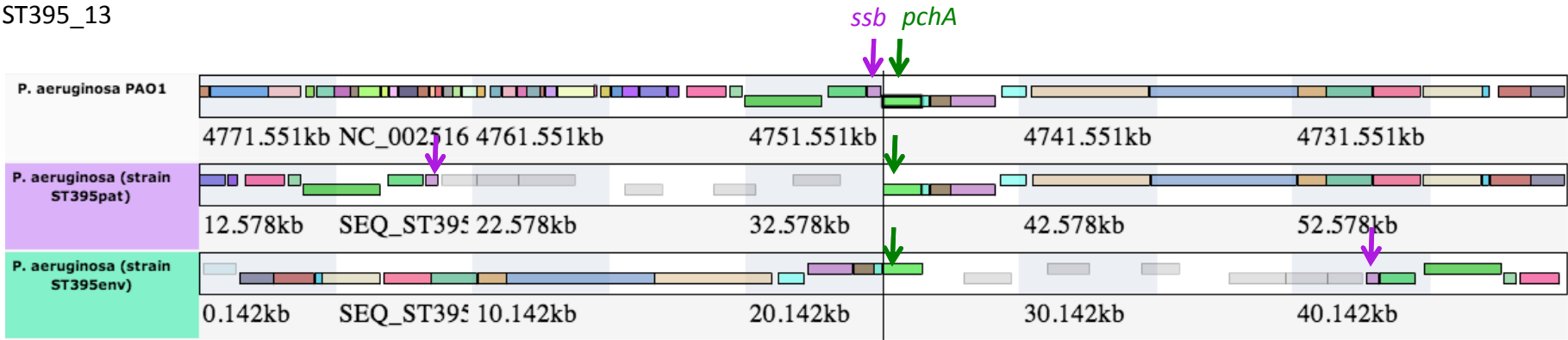

ST395\_14

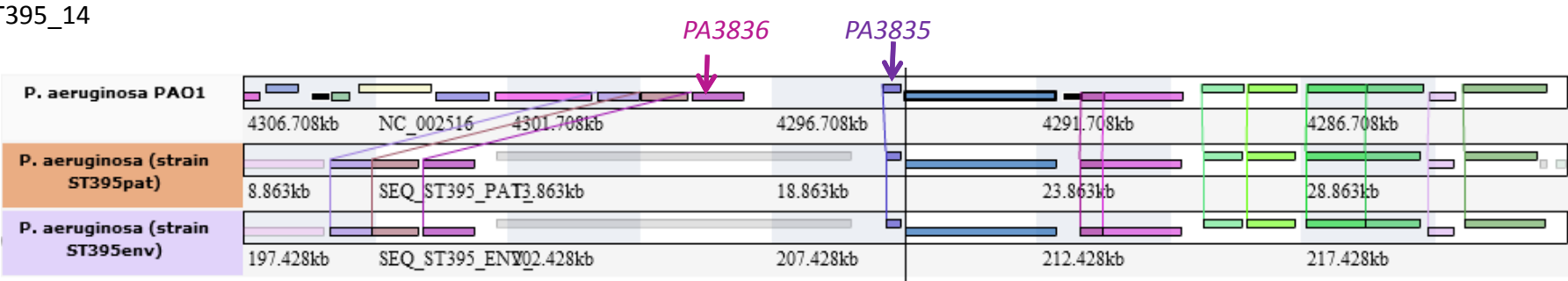

Supplement: Supplementary File 1 [file pathogens-03-00309-s001.pdf]
